# A Smart Intracellular Self‐Assembling Bioorthogonal Raman Active Nanoprobe for Targeted Tumor Imaging

**DOI:** 10.1002/advs.202304164

**Published:** 2023-09-15

**Authors:** Swati Tanwar, Behnaz Ghaemi, Piyush Raj, Aruna Singh, Lintong Wu, Yue Yuan, Dian R. Arifin, Michael T. McMahon, Jeff W. M. Bulte, Ishan Barman

**Affiliations:** ^1^ Department of Mechanical Engineering Johns Hopkins University Baltimore MD 21218 USA; ^2^ The Russell H. Morgan Department of Radiology and Radiological Science The Johns Hopkins University School of Medicine Baltimore MD 21205 USA; ^3^ Cellular Imaging Section and Vascular Biology Program Institute for Cell Engineering The Johns Hopkins University School of Medicine Baltimore MD 21205 USA; ^4^ F.M. Kirby Research Center for Functional Brain Imaging Kennedy Krieger Inc. Baltimore MD 21205 USA; ^5^ Department of Chemistry University of Science and Technology of China 96 Jinzhai Road Hefei Anhui 230026 China; ^6^ Department of Biomedical Engineering Johns Hopkins University Baltimore MD 21218 USA; ^7^ Department of Chemical & Biomolecular Engineering Johns Hopkins University Baltimore MD 21218 USA; ^8^ Department of Oncology Johns Hopkins University Baltimore MD 21231 USA

**Keywords:** Raman imaging, bioorthogonal reactions, intracellular self‐assembly, targeted imaging, prostate cancer

## Abstract

Inspired by the principle of in situ self‐assembly, the development of enzyme‐activated molecular nanoprobes can have a profound impact on targeted tumor detection. However, despite their intrinsic promise, obtaining an optical readout of enzyme activity with high specificity in native milieu has proven to be challenging. Here, a fundamentally new class of Raman‐active self‐assembling bioorthogonal enzyme recognition (nanoSABER) probes for targeted tumor imaging is reported. This class of Raman probes presents narrow spectral bands reflecting their vibrational fingerprints and offers an attractive solution for optical imaging at different bio‐organization levels. The optical beacon harnesses an enzyme‐responsive peptide sequence, unique tumor‐penetrating properties, and vibrational tags with stretching frequencies in the cell‐silent Raman window. The design of nanoSABER is tailored and engineered to transform into a supramolecular structure exhibiting distinct vibrational signatures in presence of target enzyme, creating a direct causality between enzyme activity and Raman signal. Through the integration of substrate‐specific for tumor‐associated enzyme legumain, unique capabilities of nanoSABER for imaging enzyme activity at molecular, cellular, and tissue levels in combination with machine learning models are shown. These results demonstrate that the nanoSABER probe may serve as a versatile platform for Raman‐based recognition of tumor aggressiveness, drug accumulation, and therapeutic response.

## Graphical abstract

The Raman‐active self‐assembling bioorthogonal enzyme recognition probe (nanoSABER), images the activity of aggressive tumor‐associated enzyme legumain. Owing to the presence of distinguishable vibrational modes in the cells' silent spectral region and its unique design, nanoSABER facilitates imaging legumain activity across various organizational levels with high optical contrast and sensitivity, indicating its potential for Raman‐based aggressive cancer imaging.

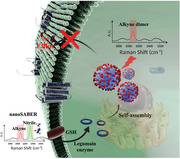

## 1 Introduction

Sensitive imaging of enzyme activity within the complex biological environment has become increasingly important for aiding in the diagnosis and prognosis of cancer, as there has been growing evidence demonstrating a close association between cancer‐cell invasion, metastasis, and angiogenesis with dysregulation of enzyme expression.^[^ ^1^, ^2^, ^3^, ^4^ ^]^ Enzymes are uniquely suited for cleaving a specific peptide bond, ubiquitous across all life forms, with extraordinary spatiotemporal control.^[^ ^5^ ^]^ This catalytic prowess has inspired the development of a new class of synthetic peptide‐based molecular imaging nanoprobes having a designed propensity for self‐assembly on interacting with specific enzymes.^[^ ^5^, ^6^, ^7^ ^]^ This strategy of injecting small sensing molecules that self‐assemble into larger nanoprobes in situ increases intracellular accumulation, reduces efflux, and permits durable signals for imaging.^[^ ^8^, ^9^, ^10^ ^]^

Among the proposed synthetic approaches, the biocompatible click condensation reaction between the 1,2‐aminothiol group (D‐cysteine) and the nitrile group of the 2‐cyanobenzothiazole (CBT) has gained tremendous attention because of the inherent advantage of intracellular self‐assembly triggered with endogenous cellular molecules under physiological conditions with a fast second‐order rate constant (9.1 M^−1^ s^−1^).^[^ ^8^ ^]^ With one enzyme being capable of converting many individual nanoprobes into self‐assembled structures, as such, this strategy leads to a large amplification of signal compared to standard labeling techniques.^[^ ^11^ ^]^ Hence, it can be employed for tailoring nanoprobes with tumor‐favorable pharmacokinetics, enhanced accumulation, and retention time in the target tissue to improve imaging contrast and drug delivery.^[^ ^5^, ^12^ ^]^ The smart intracellular self‐assembly system has been recently used in combination with various imaging modalities, such as fluorescence, nuclear, bioluminescent, and magnetic resonance imaging.^[^ ^13^, ^14^, ^15^, ^16^, ^17^, ^18^, ^19^ ^]^ Raman spectroscopy (RS), offers superior optical imaging capabilities with exquisite molecular specificity.^[^ ^20^ ^]^ The narrow spectral bandwidth of Raman peaks and their resistance to photobleaching make them more suitable for interrogating complex biological processes and quantitative measurements.^[^ ^21^ ^]^ Owing to these salient features it is now emerging as an important optical imaging technique for recording quantitative spatiomolecular maps from live and intact biological samples.^[^ ^22^, ^23^, ^24^ ^]^ However, its adoption for cancer detection has been slow due to low sensitivity and difficulty in distinguishing subtle spectral changes in the native tumor environment.^[^ ^25^ ^]^ Surface‐enhanced Raman spectroscopy, the plasmon‐enhanced counterpart of RS overcomes the sensitivity limitation by enhancing the intrinsic Raman signals by several orders of magnitude.^[^ ^26^, ^27^, ^28^ ^]^

Yet, its clinical translation has remained elusive, largely due to challenges such as in vivo toxicity, poor renal clearance, and perturbation to the native biological environment caused by metallic nanoparticles.^[^ ^21^ ^]^ We envision that the integration of RS with enzyme‐triggered intracellular self‐assembling strategies will render a paradigm of bioorthogonal Raman nanoprobes for molecular imaging across different levels of bio‐organization. In an initial proof‐of‐concept study, we demonstrated the possibility of RS in targeted imaging of furin in tumor cells and a subcutaneous xenograft model in vivo.^[^ ^29^ ^]^ However, spectral congestion between the developed nanoprobe and endogenous cellular molecules posed impediments in detection specificity in tumors. To design enzyme‐activated Raman probes suited for clinical applications, several critical issues must be addressed. First, enhanced tumor penetration must be achieved to increase accumulation in the target tumor with respect to normal tissues, and to avoid using a high dose of nanoprobe for generating detectable signals. Second, a high tumor‐to‐normal‐tissue signal ratio for generating a significant optical contrast between the nanoprobe and endogenous biomolecules. Third, the nanoprobe should leverage the multiplexing capability of RS for simultaneous imaging of multiple targets which is critical for imaging intratumoral heterogeneity.

Toward this end, we report here on the design of a smart Raman active self‐assembling bioorthogonal enzyme recognition (nanoSABER) probe as a next‐generation Raman agent for targeted tumor imaging. The nanoSABER probe presented in our current study possesses several advantages, endowing it with unparalleled potential to generate distinct signals exclusively within targeted tumors. We rationally integrated a polyarginine cell‐penetrating sequence in the nanoSABER,^[^ ^30^ ^]^ to facilitate a higher accumulation within tumors compared to normal tissues and yield discernible signals without requiring a high dose of the nanoprobe. Additionally, we have introduced vibrational tags having vibrational stretching frequency in the cell silent region (1800−2800 cm^−1^),^[^ ^31^, ^32^ ^]^ to collectively generate high optical contrast between the nanoprobe and endogenous biomolecules. Selecting legumain as a representative tumor‐associated enzyme,^[^ ^33^, ^34^, ^35^, ^36^, ^37^ ^]^ we synthesized nanoSABER with alanine–alanine–asparagine (AAN) as the legumain recognition sequence, which can produce distinct Raman signatures upon enzymatic cleavage. We demonstrate its utility for targeted imaging of legumain activity across different levels of organization from molecules to cells and tissues. Due to significant advancement in the design, nanoSABER is able to generate distinct differences in Raman signatures between legumain‐overexpressing and low‐expressing tumor cells and tissue, with the potential of Raman‐based imaging of tumor aggressiveness. Our results demonstrate the potential of nanoSABER as a broadly applicable sensing platform for a range of targets for Raman‐based tumor detection and evaluation of treatment efficacy.

## 2 Results and Discussion

### 2.1 Principle of In Vitro and In Vivo Imaging of Legumain Expression With NanoSABER

**Figure 1:**
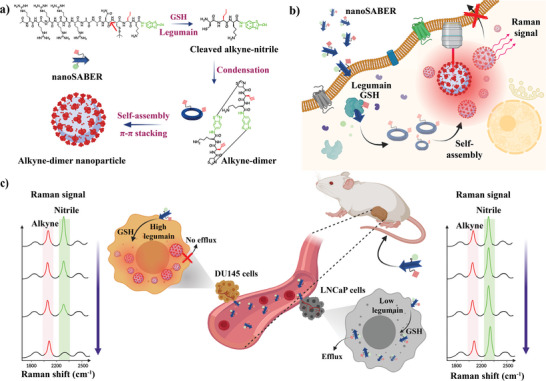
Schematic illustration for the formation of an alkyne‐dimer nanoparticle by legumain‐mediated intracellular reduction and condensation of the nanoSABER probe. a) Sequence of reaction steps showing the transformation of nanoSABER into a supramolecular self‐assembled structure. Red arrow shows the site of legumain cleavage, with the alkyne and nitrile Raman reporters shown in red and green, respectively. b) After intracellular internalization of nanoSABER in high legumain‐expressing cells (DU145 cells), it undergoes reduction by GSH and cleavage by the legumain enzyme. Alkyne‐dimers are then formed, followed by self‐assembly into alkyne‐dimer nanoparticles as a result of π–π stacking. c) Schematic illustrating the utilization of nanoSABER for targeted Raman imaging of legumain activity in DU145 and LNCaP tumor‐bearing mice. The spectral features associated with the nanoSABER probe were predominantly detected within the DU145 tumors because of legumain enzyme‐triggered intracellular self‐assembly, which also resulted in a prolonged probe retention time.

We designed the nanoSABER probe with the sequence Ac‐Arg‐Arg‐Arg‐Arg‐Arg‐Arg‐Ala‐Ala‐Asn‐Cys(StBu)‐Pra‐Lys‐CBT (R_6_‐AAN‐alkyne‐nitrile) containing three functional domains: 1) A polyarginine oligomer with six repeating units known for enhanced cell‐penetration efficiency^[^ ^30^, ^38^ ^]^; 2) The legumain substrate AAN; and 3) Raman active alkyne and nitrile groups of propargylglycine and CBT, respectively (**Figure** **1**a). The sequence of the nanoSABER probe (R_6_‐AAN‐alkyne‐nitrile) was designed such that following internalization into cells and subsequent enzymatic cleavage, as well as reduction by glutathione (GSH), the nanoSABER probe undergoes a controlled condensation reaction resulting in the formation of alkyne‐dimers (Figure 1b,c). The self‐assembly of nanoSABER into alkyne‐dimer nanoparticles is driven by the recognition of the AAN sequence, which is a unique characteristic of the legumain enzyme. Consequently, this self‐assembly phenomenon exclusively occurs in legumain‐overexpressing prostate cancer DU145 cells, where the higher expression levels of legumain enable the efficient binding and subsequent formation of alkyne‐dimer nanoparticles. Conversely, in legumain low‐expressing prostate cancer LNCaP cells, the insufficient presence of legumain limits the occurrence of self‐assembly, thereby impeding the formation of alkyne‐dimer nanoparticles (Figure 1c). We hypothesized that before condensation, the Raman signatures from both alkyne and nitrile groups would be observable. After condensation, since the nitrile group of CBT will be involved in the formation of a new thiazole ring in the cyclized alkyne‐dimer, the Raman signatures of the nitrile group will start diminishing as the reaction progresses. As a result, the ratio of alkyne/nitrile Raman peak intensity will show an increase as a function of the legumain‐assisted triggering of the condensation reaction.

### 2.2 Design and Synthesis of the NanoSABER Probe

**Figure 2:**
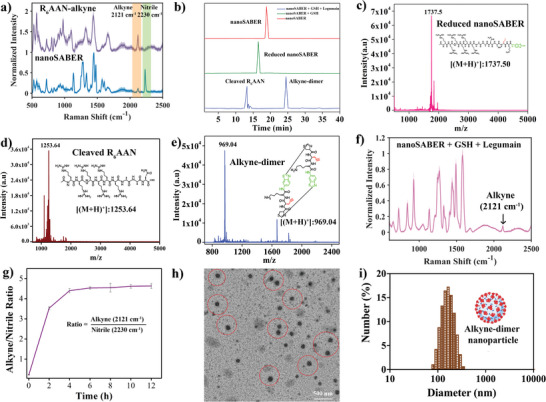
Optical and structural properties of nanoSABER (R_6_‐AAN‐alkyne‐nitrile) and alkyne‐dimer. a) Raman spectra of R_6_AAN‐alkyne and nanoSABER showing the alkyne and nitrile vibrational stretching frequency at 2121 cm^−1^ and 2230 cm^−1^, respectively, expressed as the mean ± standard deviation for n = 22 independent in vitro measurements. b) HPLC chromatogram of 250 µM nanoSABER (red) and 250 µM nanoSABER + 1 mM GSH incubated for 3 h (green), and 250 µM nanoSABER + 1 mM GSH + 3 µL (10 µg/50 µL) legumain incubated for 3 h (blue). c–e) HR‐MALDI‐TOF/MS spectra of reduced nanoSABER, cleaved R_6_‐AAN, and alkyne‐dimer. f) Raman spectra of 250 µM nanoSABER + 1 mM GSH + 3 µL (10 µg/50 µL) legumain incubated for 3 h, shown as the mean with a standard deviation of *n* = 22 independent in vitro measurements. g) Enzymatic conversion ratios of 250 µM nanoSABER in the presence of 1 mM GSH and 3 µL (10 µg/50 µL) legumain enzyme as a function of time, as calculated from the alkyne/nitrile Raman peak intensity ratio. h,i) TEM and DLS of alkyne‐dimer nanoparticles (red circles) after incubation of 250 µM nanoSABER + 1 mM GSH + 3 µL (10 µg/50 µL) legumain for 3 h at 37 °C. All subpanels reflect representative data from *i* *n vitro* experiments repeated three times unless stated.

The detailed synthetic procedures and characterization of the nanoSABER probe are given in Scheme S1 and Figures S1 and S2 (Supporting Information), respectively. The Raman signatures of nanoSABER (**Figure** **2**a) display the vibrational stretching frequencies of alkyne and nitrile at 2121 and 2230 cm^−1^, respectively. The ratio of alkyne/nitrile Raman peak intensity in the nanoSABER solutions was measured to be 0.21±0.04. Next, we studied the cleavage of the nanoSABER probe by legumain, leading to the formation of supramolecular self‐assembled nanoparticles. This was verified using high‐resolution matrix‐assisted laser desorption/ionization–time‐of‐flight mass spectrometry (MALDI), RS, and high‐performance liquid chromatography (HPLC). We first incubated 25 µM of nanoSABER probe with 250 µM GSH for 2 h at 37 °C. This reduced the disulfide bond of D‐cysteine, producing reduced nanoSABER, which was identified using HPLC and MALDI by the appearance of a peak at a retention time of 16.5 min (Figure 2b) and an observed mass of 1737.5 (Figure 2c). When nanoSABER was co‐incubated with 250 µM GSH and 3 µL (10 µg/50 µL) legumain enzyme for 3 h at 37 °C, the reduced nanoSABER that was generated in situ reacted with the legumain enzyme to form an active cleaved intermediate. The latter underwent intermolecular condensation to yield the alkyne‐dimer. The reaction mixture was analyzed using HPLC where two peaks were observed at retention times of 13.5 and 22.3 min. Both HPLC fractions were collected and characterized by MALDI. The observed mass was found to be 1253.64 (for cleaved R_6_‐AAN) and 969.04 (for alkyne‐dimer), as shown in Figure 2d,e, respectively.

We next sought to determine whether the designed nanoSABER probe shows the expected changes in Raman signal post the condensation reaction. As expected, our Raman measurements revealed that the nitrile peak at 2230 cm^−1^ disappeared (Figure 2f). To accurately evaluate the efficiency of the R_6_‐AAN‐alkyne‐nitrile nanoprobe, time‐dependent Raman measurements were performed (Figure 2g), where the ratio of alkyne/nitrile peak intensity was used as an indicator of the reaction progress. We found as the reaction progressed the alkyne/nitrile peak intensity increased from 0.22±0.01 to 4.4±0.12 and plateaued at a final value of 4.6±0.12 indicating almost complete conversion of nanoSABER to alkyne‐dimer within 3 h. These results confirm that the designed nanoSABER probe is efficiently cleaved by legumain, forming the desired supramolecular structure. To further get insight into the structural arrangements of the self‐assembled dimers, transmission electron microscopy (TEM) was performed. TEM images show the formation of alkyne‐dimer nanoparticles with an average size of 116±17 nm (Figure 2h), which can be attributed to the non‐covalent π–π stacking interaction between alkyne‐dimers.^[^ ^18^, ^39^ ^]^ The particle size distribution, as derived from TEM, is shown in Figure S3a (Supporting Information). On the contrary, the nanoSABER solution did not show any detectable nanoparticles before the addition of the legumain enzyme (Figure S3b, Supporting Information). To eliminate the possibility that the self‐assembled structures seen in TEM micrographs were due to drying effects, dynamic light scattering (DLS) measurements were performed. Which revealed particles averaging 147±20 nm in size (Figure 2i), thereby precluding the presence of drying artifacts.

Additionally, to confirm that the formation of the alkyne‐dimer was indeed induced by legumain and GSH catalysis, we designed another non‐cleavable and non‐condensable control compound R_6_‐AA‐alkyne‐nitrile (scrambled or Scr) with a sequence not having asparagine. The synthesis protocol and characterization of Scr are given in Scheme S2 and Figures S4 and S5 (Supporting Information), respectively. The ratio of alkyne/nitrile Raman peak intensity in the Scr was found to be 0.37±0.05, similar to that in nanoSABER. We hypothesized that this substrate cannot be cleaved by the legumain enzyme and therefore will not expose the D‐cysteine group for a condensation reaction to be initiated. As a result, there will be no change in the ratio of alkyne/nitrile Raman peak intensity after incubation of Scr with GSH and legumain. To validate this hypothesis, 250 µM Scr was incubated with 1 mM GSH, resulting in the reduction of the disulfide bond of D‐cysteine, as confirmed using HPLC and MALDI (Figure S6a,b, Supporting Information). After co‐incubation of Scr with 1 mM GSH and 3 µL (10 µg/50 µL) legumain for 3 h at 37 °C, there was indeed no change in the peak retention time and molecular weight indicating the Scr is non‐cleavable and non‐condensable. Furthermore, no nanoparticles could be observed in the TEM images (Figure S6c, Supporting Information).

### 2.3 In Vitro Cell Experiments

**Figure 3:**
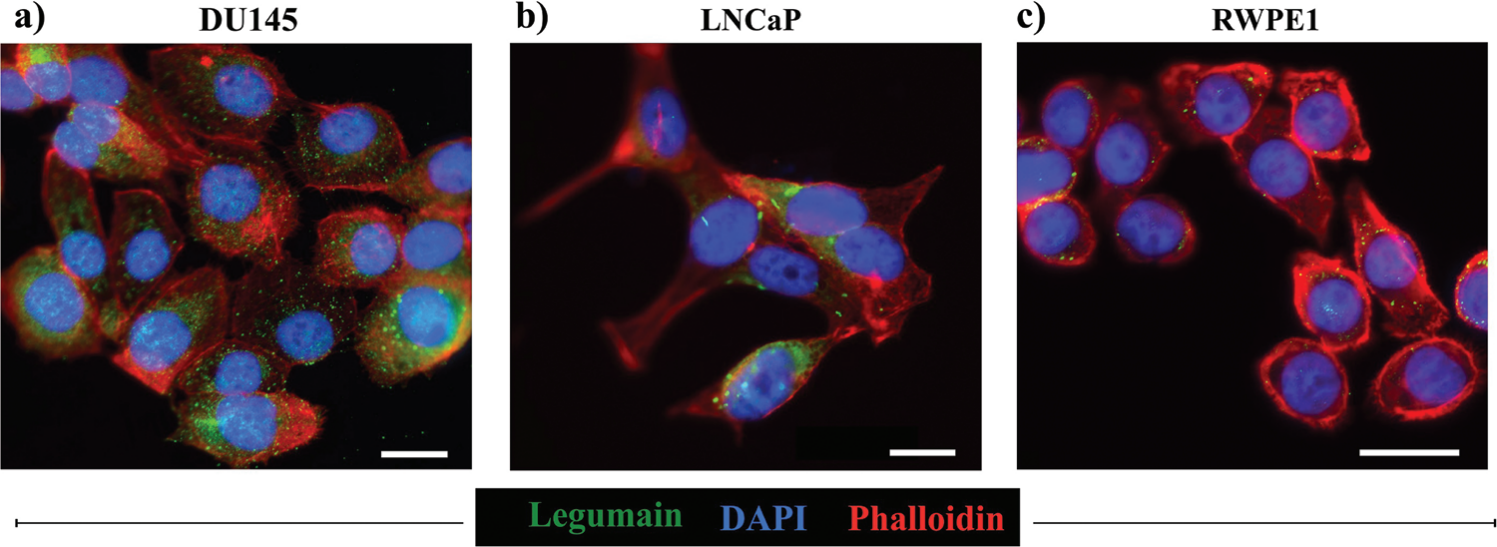
Immunofluorescence staining of legumain (green), actin filaments (phalloidin, red), and nuclei (Dapi, blue) in a) DU145 prostate tumor cells, b) LNCaP prostate tumor cells, and c) normal RWPE1 prostate cells. DU145 cells show high legumain expression compared to LNCaP and RWPE1 cells. Data are representative of three independent experiments. Scale bar = 20 µm.

To confirm that the nanoSABER nanoprobe can effectively differentiate between cells with high and low levels of legumain expression, we examined nanoSABER assembly in DU145, LNCaP, and RWPE1 cells. DU145 and LNCaP are legumain overexpressing and low‐expressing prostate cancer cell lines, respectively, whereas RWPE1 is a normal prostate epithelium cell line. Legumain‐overexpressing tumorigenic cells are aggressive in terms of migration and invasion.^[^ ^33^ ^]^ Therefore, designing new probes for targeted imaging of legumain expression is of great significance for tumor diagnosis and tumor staging. Anti‐legumain immunofluorescence imaging showed that legumain was highly expressed in DU145 cells, whereas much lower expression was observed in LNCaP and RWPE1 cells (**Figure** **3**). Compared to LNCaP and RWPE1 cells, the intracellular level of GSH in DU145 cells was significantly higher (Figure S7, Supporting Information), consistent with the previous reports.^[^ ^40^, ^41^ ^]^ A combination of both high endogenous GSH and high legumain levels is necessary in order for DU145 cells to uncage the thiol and amino groups in the nanoSABER, triggering intramolecular cyclization to form alkyne‐dimers. We performed cell viability studies to determine a biocompatible concentration of nanoSABER and found that the nanoprobe showed no toxicity up to 250 µM even after 48 h of incubation (Figure S8, Supporting Information).

**Figure 4:**
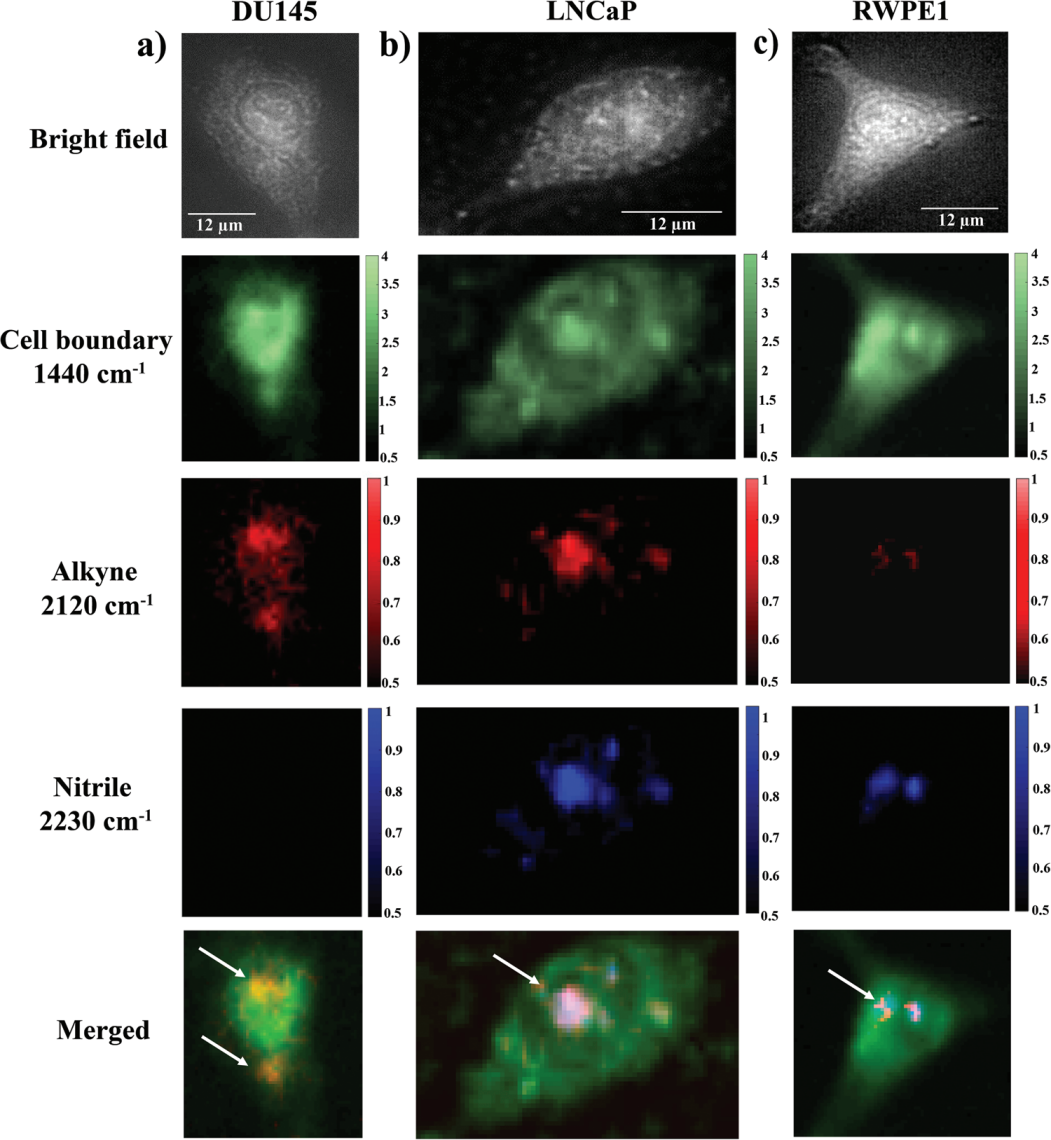
In vitro cellular Raman imaging of a) DU145, b) LNCaP, and c) RWPE1 cells incubated with 100 µM of nanoSABER solution. The 1440 cm^−1^ (green), 2120 cm^−1^ (red), and 2230 cm^−1^ (blue) signals correspond to the CH_2_ bending mode from intrinsic cellular components, and alkyne and nitrile signals from the nanoSABER probe, respectively. The merged image shows an overlay of endogenous cellular signals, alkyne, and nitrile Raman signals. Data are representative of three independent experiments.

Raman measurements were performed on cells cultured on quartz slides. Cells were incubated with 100 µM nanoSABER probe solution for 3 h, then fresh media was added and incubation was continued for an extra 30 min. Subsequently, the cells were washed three times with phosphate‐buffered saline (PBS), fixed with 4% paraformaldehyde (PFA) for 20 min, and washed with PBS again. Finally, cells were imaged using a confocal Raman microscope. Cell boundaries were constructed using the Raman peak at 1440 cm^−1^ characteristic of intrinsic cellular signals.^[^ ^29^ ^]^ Confocal Raman images of DU145 cells showed the presence of an alkyne signal at 2120 cm^−1^ distributed throughout the intracellular region with undetectable signal levels at 2230 cm^−1^ corresponding to the nitrile group of the nanoSABER nanoprobe (**Figure** **4**a). These spectral features indicate legumain‐mediated intracellular self‐assembly of nanoSABER into alkyne‐dimer nanoparticles in DU145 cells. The Raman mapping results were consistent with our in vitro solution‐based measurements (Figure 2f) where the nitrile peak diminished after the formation of alkyne‐dimer.

To further confirm that the transformation of nanoSABER into alkyne‐dimer nanoparticles is indeed induced by legumain, we incubated DU145 cells with a scrambled (Scr) sequence. The Raman map of DU145 cells showed the presence of both alkyne and nitrile peaks (Figure S9, Supporting Information) as intracellular legumain is not able to recognize and cleave the Scr nanomotif. Further, the absence of alkyne and nitrile peaks from the confocal Raman map of only DU145 cells shows that peaks at 2120 and 2230 cm^−1^ are specific to the designed nanoprobe and only appear when the nanoprobe is internalized and not found in cells in normal conditions (Figure S10, Supporting Information). When LNCaP and RWPE1 cells were incubated with the nanoSABER, signals for both alkyne and nitrile groups from the nanoprobe were observed (Figure 4b,c). This provides evidence that the nanoSABER self‐assembles into alkyne‐dimer nanoparticles only in legumain‐overexpressing prostate cancer DU145 cells. The overall lower intensity of the Raman signal in RWPE1 cells compared to that of LNCaP and DU145 cells may be attributed to the interaction between the polyarginine peptides and the cell membrane, since the cancer cell membrane has a more negative charge from anion lipids leading to a more efficient penetration compared to normal cells.^[^ ^42^ ^]^ Taken together, these results indicate that the designed nanoSABER Raman probe is specific for imaging legumain activity with excellent sensitivity.

**Figure 5:**
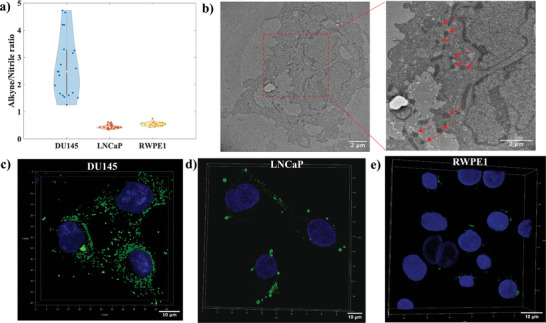
a) Ratio of the alkyne (2120 cm^−1^) to nitrile (2230 cm^−1^) Raman peak intensity obtained by Raman measurements performed on cell pellets of DU145, LNCaP, and RWPE1 cells incubated with nanoSABER. b) TEM image of DU145 cells incubated with 100 µM of nanoSABER solution, inset shows high magnification TEM image of the red rectangle area. Red arrows indicate regions featuring alkyne‐dimer nanoparticles. c–e) Confocal microscopy images of c) DU145, d) LNCaP, and e) RWPE1 cells incubated with 3 µM Alexa‐nanoSABER solution. Green fluorescence denotes legumain‐mediated, self‐assembled Alexa‐alkyne‐dimer nanoparticles. Cell nuclei were counterstained with DAPI (blue). All subpanels reflect representative data from *in vitro* experiments repeated three times.

To get a more quantitative assessment, we performed Raman measurements on cell pellets of DU145, LNCaP, and RWPE1 cells incubated with nanoSABER. We found a significant increase in the ratio of the alkyne to nitrile Raman peak intensity for DU145 cells ranging from 1.5 to 4.5 (**Figure** **5**a). This differential ratio can be attributed to different levels of expression of legumain within a cell population and is representative of cell‐to‐cell variability. Even the lowest obtained ratio of 1.5 is considerably higher than the highest ratio for both LNCaP and RWPE1 indicating that the nanoprobe can detect even a very low level of legumain expression.

We further characterized the intracellular self‐assembly of the nanoprobe with TEM and confocal fluorescence microscopy. TEM indicated that the alkyne‐dimer nanoparticles were self‐assembled inside DU145 cells (Figure 5b). For fluorescence studies, Alexa 488 conjugated nanoSABER nanoprobes were utilized. The synthesis protocol and characterization of Alexa‐nanoSABER are given in Scheme S3 and Figures S11–S13 (Supporting Information), respectively. Confocal microscopy of DU145, LNCaP, and RWPE1 cells incubated with Alexa‐nanoSABER showed a similar pattern as observed with confocal Raman imaging (Figure 5c–e). An abundant intracellular aggregation of green fluorescent Alexa‐alkyne‐dimer nanoparticles was observed throughout the cytoplasm of DU145 cells, but much less in LNCaP and near‐absent in RWPE1 cells. All three cell lines showed negligible fluorescence after incubation with Alexa 488 dye alone (Figure S14, Supporting Information).

### 2.4 In Vivo and *Ex Vivo* Raman Imaging of Legumain Activity with the NanoSABER Probe

**Figure 6:**
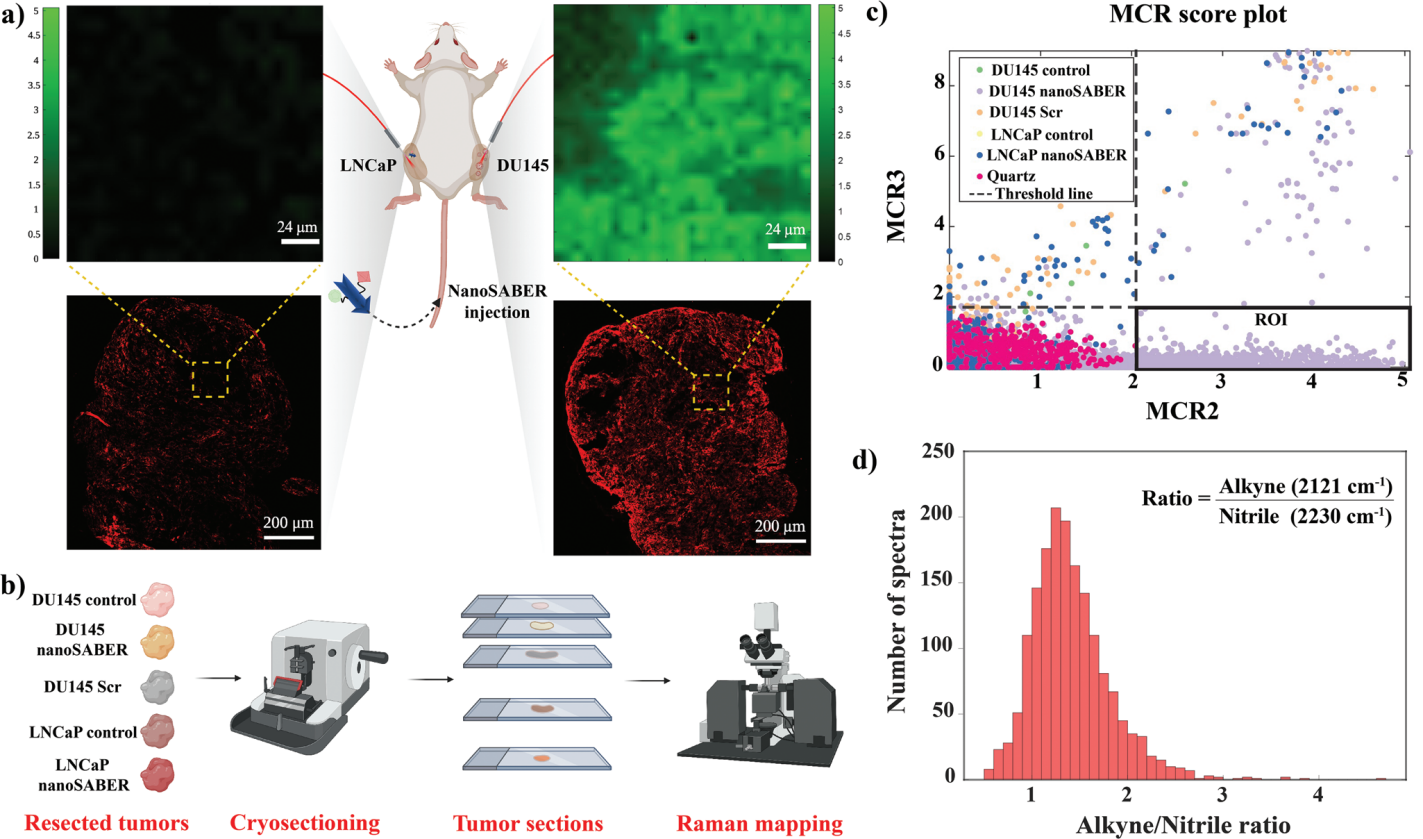
a) In vivo Raman imaging of DU145 and LNCaP tumor‐bearing mice 2 h post IV injection of nanoSABER. The green color represents the Raman signal corresponding to the alkyne peak at 2120 cm^−1^. For illustration purposes, contralateral tumors within the same mice are shown. The bottom panel shows fluorescence images showing legumain expression. b) Schematic outline of *ex vivo* tumor experiments. Excised tumors were cryosectioned, placed on quartz slides, and scanned with a HORIBA Raman microscope. c) Score plot of MCR2 versus MCR3 for the two tumor cell lines. The applied thresholds (dashed lines) for the MCR2 and MCR3 components ensure that the ROI (lower right quadrant) is free from the background spectral signal. d) The ratio of alkyne to nitrile Raman peak intensity of all spectra within the ROI. The subpanels showcase representative data from three independent mice in each experimental group.

To further evaluate the ability of the nanoSABER probe to delineate legumain‐overexpressing tumors, in vivo Raman imaging of tumors of mice inoculated with DU145 and LNCaP cells was performed (**Figure** **6**a). The Raman images were captured 2 h after the intravenous (IV) injection of the nanoSABER probe. Intense Raman signal corresponding to the alkyne peak at 2120 cm^−1^ can be observed for the entire DU145 tumor, indicative of the effective intracellular self‐assembly of nanoSABER probe into alkyne‐dimer nanoparticles. The signal intensity within the LNCaP tumors was significantly weaker, indicating a lower accumulation of alkyne‐dimer nanoparticles. Our results validated that nanoSABER experienced tumor legumain‐triggered self‐assembly into alkyne‐dimer nanoparticles that effectively generated distinct and higher Raman signals in DU145 tumors. To validate our findings, we included a control group that received a PBS injection instead of the nanoSABER probes. In both types of tumors, no Raman signal was detected in the control group (Figure S15, Supporting Information), further confirming that the observed signals in the experimental groups were indeed attributable to the legumain‐instructed self‐assembly of alkyne‐dimer nanoparticles.

*Ex vivo* Raman imaging was conducted on the tumor excised from the tumor‐bearing mice at 2 h post‐administration of nanoSABER (Figure 6b–d). To distinguish the probe signal from endogenous autofluorescence and Raman‐active molecules in the tissue matrix, multivariate curve resolution (MCR) analysis was performed on the spectral dataset recorded from the Raman‐mapped tumor sections. MCR is designed to tackle such problems by expressing the original data through a bilinear model of pure component meaningful contributions.^[^ ^43^ ^]^ Tissue sections were divided into five groups: DU145 control, DU145‐nanoSABER, DU145‐Scr, LNCaP control, and LNCaP‐nanoSABER, with the control group representing mice injected with 100 µL of PBS vehicle. Collected Raman spectra were divided into the above‐mentioned classes. The tumor dataset was also supplemented with spectra from the quartz coverslip and a separate class was created to preclude the impact of background spectral features in the ensuing analysis. Figure S16a (Supporting Information) shows seven component spectra following MCR analysis. Visual inspection of these reveals that while MCR1 is influenced by the quartz signal, MCR2‐MCR7 shows tissue‐specific features. Crucially, MCR2 shows distinct alkyl and nitrile peaks of 1800–2800 cm^−1^ in the cell‐silent region (Figure S16b, Supporting Information) indicating the presence of the nanoSABER probes. Since we are particularly interested in the wavenumber features in the cell‐silent region (i.e., the nanoSABER‐specific bands in the otherwise biologically silent spectral region), MCR2 serves as the principal differentiator.

To create a readily interpretable two‐dimensional (2D) visualization plot, we plotted MCR2 scores against that of MCR3, which contains primarily tissue‐characteristic and background features (Figure 6c). Our aim was to identify the region of interest (ROI) that displays alkyne and nitrile features in a cell‐silent area with minimal interference from endogenous tissue background signal. The highest MCR2 and MCR3 scores for the quartz‐only spectra were used as the lower and upper thresholds, respectively, to depict significant contributions from the individual components. The 2D plot demonstrates that spectra in the lower right quadrant region of interest are only found in mice with DU145 tumors that received the nanoSABER probe. This is indicative of a 100% specificity of the nanoSABER probe, without secondary non‐specific spectra from any of the other tumor treatment classes in the ROI. Moreover, 15% of all spectra obtained from mice with DU145 tumors treated with the nanoSABER probe were situated within the ROI, which confirms the high penetration efficiency of the nanoprobes. These results underscore the promise of this platform in the context of the considerable challenges in delivery efficiency with a recent meta‐analysis revealing that only 0.7% (median) of the administered nanomedicinal dose is correctly delivered to targeted sites.^[^ ^44^ ^]^

The average ratio of alkyne to nitrile Raman peak intensity was found to be 1.5, which is consistent with our legumain enzyme solution and cell‐based measurements (Figure 6d). This observation is attributed to the self‐assembly of nanoSABER into alkyne‐dimer nanoparticles due to the high expression level of legumain in DU145 tumors. Our results indicate that our designed nanoprobe exhibits unique spectral features upon internalization in DU145 tumors facilitating its detection with high sensitivity and selectivity even within the normal tumor microenvironment.

### 2.5 Toxicity and Biocompatibility Evaluation of NanoSABER

**Figure 7:**
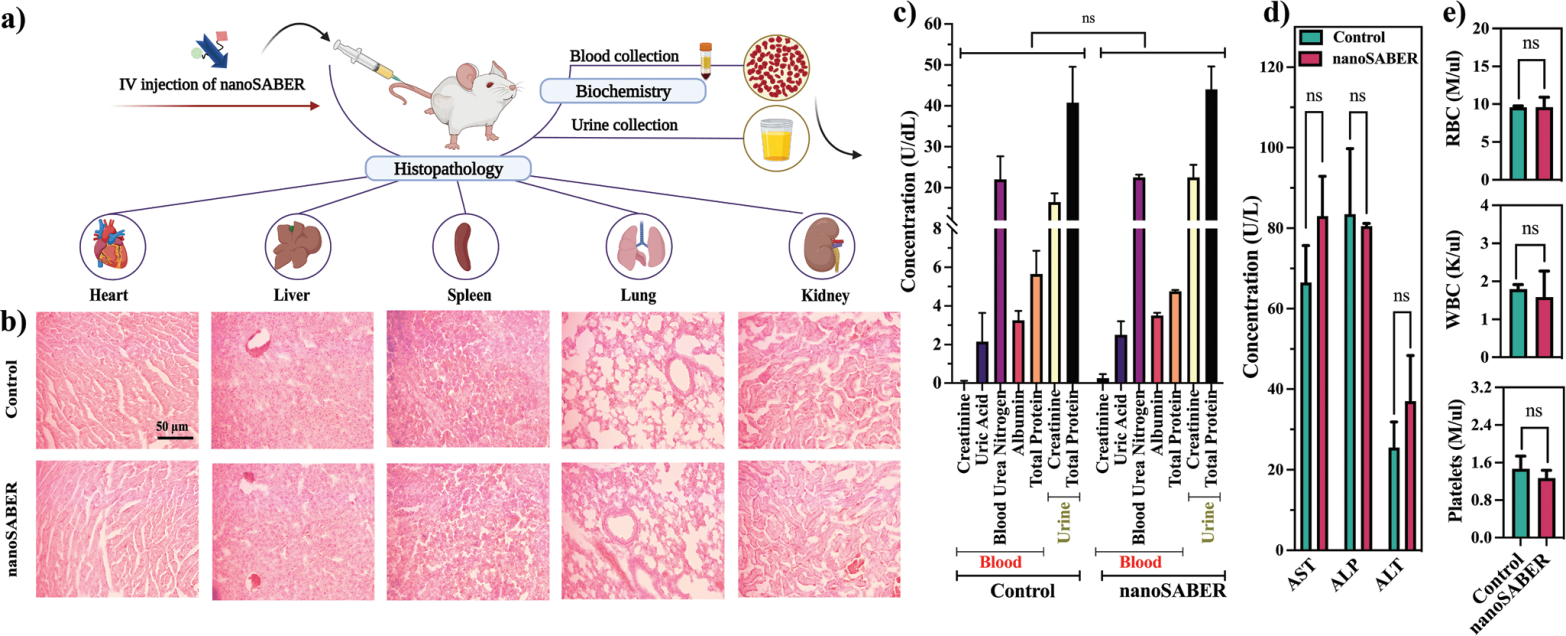
a) Schematic illustration of in vivo toxicity and biocompatibility assessment 7 days after IV injection of nanoSABER. b) Histology of major organs excised and stained with H&E for PBS (control) or nanoSABER injection. c) Kidney function test for blood plasma and urine. d) Liver function test for the enzymes aspartate transaminase (AST), alkaline phosphatase (ALP), and alanine transaminase (ALT) in blood plasma. e) Bone marrow function test for red blood cells (RBCs), white blood cells (WBCs), and platelets in blood plasma. All data are presented as mean ± standard deviation, *n* = 3. *p*‐Values of <0.001 (***), 0.001 to 0.01 (**), and 0.01 to 0.05 (*) were considered significant.

The in vivo toxicity and biocompatibility of nanoSABER were investigated following IV injection of nanoSABER in mice (**Figure** **7**a). Hematoxylin and eosin (H&E) staining of major organ tissues revealed no signs of histopathological damage or tissue inflammation for the nanoSABER‐injected group (Figure 7b). In addition, we examined various common indicators related to renal function (Figure 7c), liver function (Figure 7d), and bone marrow function (Figure 7e). In comparison to the PBS group, all the parameters measured in the nanoSABER‐treated group fell within a normal reference range. These findings further confirm the biocompatibility and safety of the nanoSABER for in vivo targeted tumor imaging.

## 3 Conclusion

In summary, by leveraging a combination of CBT‐cysteine click condensation chemistry and Raman reporters active in the cell silent region we designed an enzyme‐responsive intracellular self‐assembling Raman imaging nanoprobe for targeted imaging of tumor‐associated enzymatic activity in tumor cells and a xenograft mouse model. The nanoSABER nanoprobe was responsive to legumain enzyme activity and showed two distinct peaks in the cell silent region at 2120 cm^−1^ and 2230 cm^−1^. It undergoes a rapid intramolecular condensation and self‐assembly in the legumain overexpressing microenvironment and the peak at 2230 cm^−1^ corresponding to the nitrile group of CBT diminished as it was involved in the formation of a new thiazole ring. The developed nanoSABER probes exhibit distinct Raman signatures after internalization in DU145 and LNCaP cellular environments having high and low levels of legumain expression, respectively. The Raman imaging results in DU145 tumor‐bearing mice showed similar patterns as found in the solution and cell‐based measurements. The enzyme‐triggered self‐assembling approach presented in this research enhanced the intracellular accumulation of Raman probes in targeted DU145 tumor cells due to in situ probe assembly, generating enhanced and long‐lasting Raman signals. Furthermore, as a result of the innate specificity arising from the easily distinguishable vibrational modes in the cell silent spectral region, the nanoSABERs offered a high signal‐to‐noise ratio making Raman‐based imaging of enzyme activity a reality. Our results suggest that the nanoSABER Raman probe can serve as a general molecular platform for Raman‐based imaging of enzyme activity by simply changing the enzyme‐responsive sequence in the nanoprobe, which could facilitate the development of other activatable probes and contribute to tumor detection and treatment efficacy evaluation. Furthermore, with the advances in the availability of a versatile palette of novel Raman reporters with narrower full width at half maximum of spectral peaks/vibrational modes,^[^ ^45^, ^46^, ^47^ ^]^ the designed platform may pave the way toward Raman‐based multiplexed molecular imaging, opening up new imaging opportunities.

We envision that the approach should ultimately find its utility in multiplexed biomolecular activity imaging surpassing the detection ability of current fluorescent probes. In a clinical scenario, the developed approach may facilitate the imaging of cancer‐specific molecular targets, enabling the early diagnosis of aggressive tumors and improving treatment outcomes.

## 4 Experimental Section

### Materials and General Methods

All materials were sourced from Sigma, Chem Impex, or Thermo Fisher and used as received, with purification performed only when explicitly indicated. Recombinant human legumain was purchased from R&D systems. Milli‐Q water was used in all experiments. All peptides were synthesized using a solid‐phase peptide synthesis (SPPS) method with 2‐chlorotrityl chloride (CTC) resins on AAPPTec focus Xi and focus XC multi‐peptide synthesizers. The mass of synthesized peptides was determined using Bruker AutoFLex III Maldi‐TOF or Voyager DE‐STR MALDI‐TOF mass spectrometers. ^1^H NMR spectra were obtained on a 400 MHz Bruker Avance at 27 °C in dimethylsulfoxide (DMSO)‐d_6_. Ultraviolet–visible (UV–Vis) absorption spectra were recorded with a Perkin Elmer Lambda 950 UV–Vis spectrophotometer. Reverse‐phase high‐performance liquid chromatography (RP‐HPLC) analysis was performed on a Shimadzu CBM‐40 system using a C18 column and a Waters Breeze RP‐HPLC system equipped with a Waters XBridge peptide BEH C18 column using acetonitrile (CH_3_CN) 0.1% of trifluoroacetic acid (TFA) in water as the eluent. DU145, LNCaP, and RWPE1 cells were obtained from ATCC and cultured at 37 °C and 5% CO_2_ in a humidified atmosphere. DU145 and LNCaP cells were cultured in Roswell Park Memorial Institute (RPMI) 1640 media with 10% fetal bovine serum and 100 U mL^−1^ of penicillin‐streptomycin. For RWPE1 cells, serum‐free keratinocyte SFM supplemented with human recombinant epidermal growth factor and bovine pituitary extract was added as provided by the supplier. Raman measurements were performed using an XploRA PLUS Raman microscope (HORIBA Instruments Inc.). TEM images were acquired using an FEI Talos 200SC FEG electron microscope at an operating voltage of 200 kV. High‐resolution fluorescence imaging was performed using a Leica SP8 confocal microscope equipped with a 63× oil‐immersion objective. The size distribution of nanoparticles was determined using dynamic light scattering (DLS) (Zetasizer Nano, Malvern Instruments) at room temperature (RT). Cell counting was done with a Countess 3 (Invitrogen) automated cell counter.

### In Vitro Raman Imaging

Raman spectra of all synthesized peptides were acquired using a 785 nm laser and a 50× objective. The acquisition time was 10 s with 5 repetitions. All spectra were processed using custom‐written scripts in MATLAB for a tenth‐order least‐squares polynomial baseline correction with a moving window of 50. All Raman cellular imaging maps were acquired using 785 nm laser excitation and a 60× water immersion objective. For each acquisition time, the number of accumulations were 6 s and 8 acquisitions, respectively. For sample preparation, 1 × 10^5^ cells were cultured in six‐well plates containing 10×10 mm quartz slides for 24 h. Cells were incubated with 100 µM of nanoSABER or Scr nanoprobe for 3 h followed by replacement with fresh media and additional incubation for 30 min. Cells were washed with PBS three times and then fixed with 4 % paraformaldehyde (PFA) for 20 min, resuspended back in PBS, and then imaged using a confocal Raman microscope. All spectra were processed using custom‐written scripts in MATLAB for a tenth‐order least‐squares polynomial baseline correction with a moving window of 5. For cell pellet experiments, after incubation of 5 × 10^6^ cells with 100 µM nanoSABER in culture medium for 3 h at 37 °C, the medium was replaced with fresh media without nanoSABER and cells were incubated for an additional 30 min. After washing with PBS, cells were trypsinized and the cell pellet was collected by centrifugation at 200 g for 5 min. Cells were washed with PBS three times and the pellet was dried on an aluminum plate. For each group, three independent experiments were performed, and from each pellet, we recorded Raman spectra from eight different positions. Raman spectra were obtained using a 785 nm laser and 50 × objective lens. For each spectrum, five acquisitions with a duration of 10 s were acquired.

### Glutathione (GSH) Detection

A cellular GSH detection assay Kit (13859, Cell signaling technology) was used for cellular GSH detection according to the manufacturer's protocol. Briefly, cell extracts were transferred to a 96‐well optical bottom assay plate, mixed with assay buffer, and incubated for 60 min at RT. The reaction products were measured with a plate reader at an excitation wavelength of 380 nm and an emission wavelength of 485 nm. A standard calibration curve of reduced GSH was used to determine the amount of GSH in the samples.

### Immunostaining

Cells were cultured on chamber slides to reach 60% confluency. Cells were washed twice with PBS, then fixed and permeabilized with 4% PFA/0.1% Triton X‐100 for 20 min. Cells were washed three times with PBS, blocked with 2% BSA in PBS for 1 h at RT, and subsequently incubated with an anti‐legumain antibody (ab244251, 1:200, Abcam) overnight at 4 °C. Cells were washed 3 times with PBS and incubated with Alexa fluor 488 IgG secondary antibody (1:250) and Alexa Fluor 594 phalloidin at RT for 45 min. Cells were embedded and coverslipped with 4,6‐diamidino‐2‐phenylindole (DAPI) mounting medium and visualized using a Zeiss Axiovert 200 M inverted epifluorescence microscope.

### Cell Uptake and Viability Studies

For cell viability testing, the total numbers of DU145, LNCaP, and RWPE1 cells were counted after staining with Trypan blue using an Invitrogen Countess 3 automated cell counter. Cells were incubated with nanoSABER at different concentrations. Cell viability was calculated as a percentage of control (untreated cells) set at 100%. Data were expressed as mean ± standard deviation. from three independent experiments.

### In Vivo Raman Imaging of Prostate Tumors

All animal experiments were approved by an institutional animal care and use committee (IACUC) protocol. NU/J nude mice (male, 6–8 weeks old) were subcutaneously injected with 1 × 10^6^ DU145 or LNCaP cells in the right flank. Once the tumor reached a volume of 100–200 mm^3^, mice were IV injected with 0.0125 mmol kg^–1^ nanoSABER or PBS (control group). After 2 h, Raman imaging was performed to determine the amount of intracellular self‐assembly within the tumors.

### Ex vivo Raman Imaging of Prostate Tumors

All animal experiments were carried out under an approved IACUC protocol. NU/J nude mice (male, 6–8 weeks) were subcutaneously injected with 1×10^6^ DU145 and LNCaP cells in the right flank. Once the tumor reached a volume of 100–200 mm^3^, mice were IV injected with 0.0125 mmol kg^–1^ nanoSABER and Scr nanoprobe (dissolved in PBS with 5% DMSO). After 2 h, the nanoprobe‐injected mice were sacrificed and the tumor was removed and placed in PBS. Tumors were frozen in optical cutting temperature medium and cryosectioned into 12 µm slices, and then transferred to oxygen plasma‐cleaned quartz slides for confocal Raman microscopic imaging.

### In Vivo Biocompatibility Assays

All animal experiments were carried out under an approved IACUC protocol. The biocompatibility of nanoSABER was evaluated in vivo, using healthy female rag2 mice that were divided into two groups (*n* = 3 each). The first group was IV injected with PBS (100 µL, pH = 7.4) as control, while the second group was IV injected with nanoSABER (100 µL, 0.0125 mmol kg^–1^). All mice were euthanized 7 days post‐injection. Major organs (heart, liver, lung, kidney, and spleen) were harvested and fixed with 4% PFA for histological analysis by hematoxylin and eosin (H&E) staining. Various indicators of kidney and liver function were examined. These were creatinine, uric acid, blood urea nitrogen, albumin, total protein for kidney function and enzymes aspartate transaminase (AST), alkaline phosphatase (ALP), alanine transaminase (ALT) for liver function. Blood samples were also collected for hematological analysis. The tested blood parameters include red blood cell (RBC), white blood cell (WBC), and platelet counts.

### Statistics and Reproducibility

Data were presented as means ± standard deviation (s.d.). Statistical analysis was conducted using either a one‐way analysis of variance (ANOVA), one‐way ANOVA with Tukey's HSD multiple comparisons post‐hoc test, or two‐tailed Student's t‐test. The significance levels for these tests were set as follows: *p<0.05, **p<0.01, and ***p<0.001. To ensure sufficient statistical power (>80%, *p* = 0.05) for determining the expected effect sizes, sample sizes were carefully chosen based on preliminary data or previous experience with similar experiments. The number of independent repetitions for each experiment was indicated in the figure captions. All statistical calculations were performed using Matlab or GraphPad Prism v.9.0.0. For Raman spectral analysis, all spectra were processed using custom‐written scripts in MATLAB for a tenth‐order least‐squares polynomial baseline correction with a moving window of 5.

## Conflict of Interest

The authors declare no conflict of interest.

## Supporting information

Supporting Information

## Data Availability

Research data are not shared.
