# Supplementary material for: A Smart Intracellular Self‐Assembling Bioorthogonal Raman Active Nanoprobe for Targeted Tumor Imaging
Source: Adv Sci (Weinh). 2023 Sep 15;10(34):2304164. doi: 10.1002/advs.202304164 (PMC10700673; doi:10.1002/advs.202304164)
Supplement: Supplementary file 1 — Supporting Information [file ADVS-10-2304164-s001.pdf]

ADVANCED SCIENCE
Open Access

Supporting Information

for *Adv. Sci.*, DOI 10.1002/advs.202304164

A Smart Intracellular Self-Assembling Bioorthogonal Raman Active Nanoprobe for Targeted Tumor Imaging

Swati Tanwar, Behnaz Ghaemi, Piyush Raj, Aruna Singh, Lintong Wu, Yue Yuan, Dian R. Arifin, Michael T. McMahon, Jeff W. M. Bulte and Ishan Barman**

Supporting Information

A smart intracellular self-assembling biorthogonal Raman active nanoprobe for targeted tumor imaging

Swati Tanwar, Behnaz Ghaemi, Piyush Raj, Aruna Singh, Lintong Wu, Yue Yuan, Dian R. Arifin, Michael T. McMahon, Jeff W.M. Bulte, Ishan Barman**

1. Peptide synthesis and characterization

Synthesis of nanoSABER (R_6AAN-alkyne-nitrile) with the sequence Ac-Arg-Arg-Arg-Arg-Arg-Arg-Ala-Ala-Asn-Cys(StBu)-Pra-Lys-CBT:

Scheme S1. Synthetic route for nanoSABER.

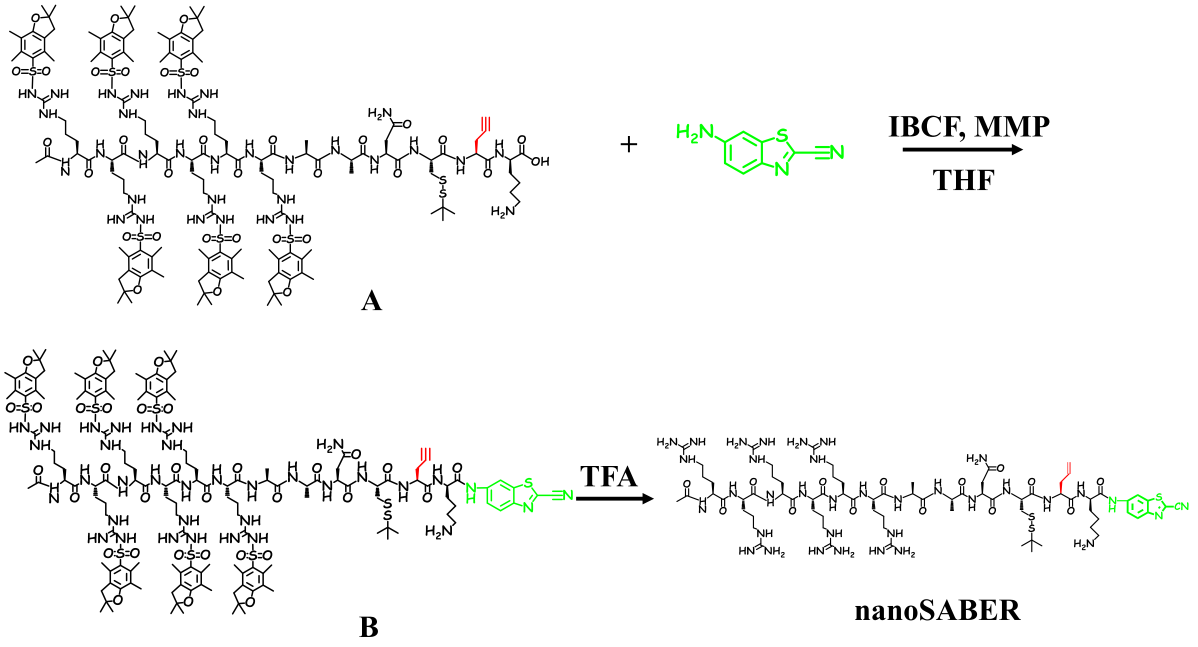

Synthesis of B: The peptide Ac-[Arg(Pbf)]$_6$-Ala-Ala-Asn-Cys(StBu)-Pra-Lys(Boc)-COOH (A) was synthesized with the SPPS method on CTC resins. After sequential washing with N,N-**Dimethylformamide** (DMF), and dichloromethane (DCM), resins were treated with 2% TFA/DCM to cleave the peptide from the resin without removing the protecting groups. Compound A was produced after concentrating the solvent using a rotary evaporator followed

by recrystallization with diethyl ether. Isobutyl chloroformate (IBCF, 20.48 mg, 0.15 mmol) was added to a mixture of compound A (500 mg, 0.15 mmol) and 4-methylmorpholine (MMP, 30.3 mg, 0.3 mmol) in tetrahydrofuran (THF,10.0 mL) at 0 °C under N_2 gas atmosphere. The reaction mixture was stirred for 40 minutes. A solution of 2-cyano-6-aminobenzothiazole (CBT, 31.5 mg, 0.18 mmol) and additional IBCF (6.8 mg, 0.05 mmol) was added to the reaction mixture with continued stirring for 1 h at 0 °C, then stirred overnight at RT. The synthesized compound B (400 mg) was precipitated from the reaction mixture using diethyl ether, dried, and used for further steps.

Synthesis of nanoSABER: The Boc and Pbf protecting groups of compound B were cleaved with a cleavage cocktail having 95% TFA, 4% DCM, and 1.0% triisopropylsilane (TIPS) for 4 h. The nanoSABER (40 mg, yield: 27%) was obtained after RP-HPLC purification using water-acetonitrile added with 0.1% TFA as the eluent (from 80:20 to 20:80). MS: calculated for nanoSABER $[(M+H)^+]$: m/z 1825.23; observed MALDI-TOF/MS: m/z 1825.63 (Figure S1).

^{1}H NMR of nanoSABER ($C_{74}H_{125}N_{35}O_{14}S_3$, d_6-DMSO, 400 MHz, Figure S2): δ 7.55 (dd, *J* = 10.5, 2.0 Hz, 12H), 7.13 (d, *J* = 8.0 Hz, 10H), 7.12 – 6.82 (m, 102H), 6.58 (ddd, *J* = 16.0, 10.0, 7.9 Hz, 132H), 3.34 (dt, *J* = 10.6, 6.6 Hz, 10H), 3.20 (t, *J* = 9.3 Hz, 16H), 3.03 (d, *J* = 6.7 Hz, 101H), 1.91 (d, *J* = 29.7 Hz, 173H), 1.75 – 1.34 (m, 107H), 1.12 (dd, *J* = 3.6, 1.9 Hz, 5H), 0.65 (d, *J* = 6.7 Hz, 35H), 0.45 (s, 65H), 0.29 (s, 245H), 0.06 (s, 89H), 0.01 – -0.07 (m, 108H).

Synthesis of Ac-Arg-Arg-Arg-Arg-Arg-Arg-Ala-Ala-Cys(StBu)-Pra-Lys-CBT (Scr):

Scheme S2. Synthetic route for Scr.

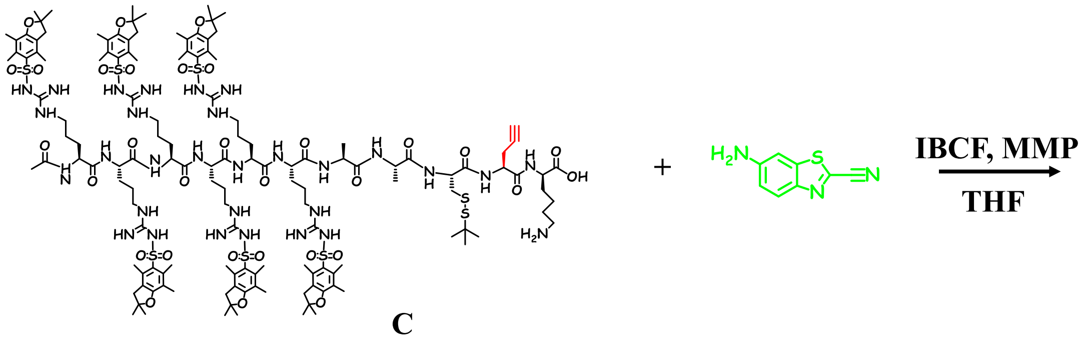

C

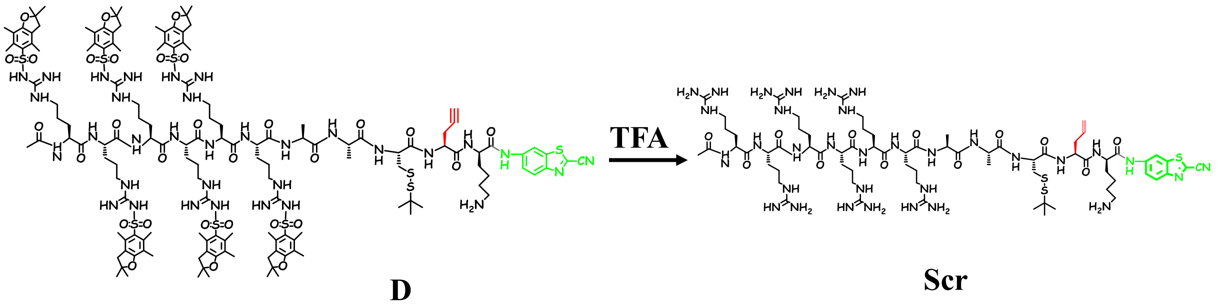

D **Scr**

Synthesis of C: The peptide Ac-[Arg(Pbf)]$_6$-Ala-Ala-Cys(StBu)-Pra-Lys(Boc)-COOH (C) was synthesized with the SPPS method on CTC resins. After sequential washing with DMF and DCM, resins were treated with 2% TFA/DCM to cleave the peptide from the resin without removing the protecting groups. Compound C was produced after concentrating the solvent using a rotary evaporator followed by recrystallization with diethyl ether. IBCF (20.48 mg, 0.15 mmol) was added to a mixture of compound C (500 mg, 0.15 mmol) and MMP (30.3 mg, 0.3 mmol in THF (10.0 mL) at 0 °C under N_2 gas atmosphere. The reaction mixture was stirred for 40 minutes. A solution of CBT (31.5 mg, 0.18 mmol) and additional IBCF (6.8 mg, 0.05 mmol) was added to the reaction mixture with continued stirring for 1 h at 0°C, then stirred overnight at RT. The synthesized compound D (450 mg) was precipitated from the reaction mixture using diethyl ether, dried, and used for further steps.

Synthesis of Scr: The Boc and Pbf protecting groups of compound D were cleaved with a cleavage cocktail having 95% TFA, 5% DCM, and 1% TIPS for 4 h. Scr (36 mg, yield: 30%) was obtained after RP-HPLC purification using water-acetonitrile added with 0.1% TFA as the eluent (from 80:20 to 20:80). MS: calculated for Scr [$(M+H)^+$]: m/z 1711.12; observed MALDI-TOF/MS: m/z 1711.65 (Figure S4a). Raman spectrum of Scr is given in Figure S4b.

^{1}H NMR of Scr ($C_{70}H_{119}N_{33}O_{12}S_3$, d_6-DMSO, 400 MHz, Figure S5): ^{1}H NMR (400 MHz, DMSO) δ 10.65 (s, 3H), 8.76 (s, 4H), 8.39 (d, *J* = 7.9 Hz, 6H), 8.16 (t, *J* = 24.1 Hz, 27H), 7.91

(dd, J = 79.6, 8.0 Hz, 30H), 7.67 – 6.73 (m, 72H), 4.47 (dd, J = 42.0, 6.5 Hz, 12H), 4.20 (d, J = 29.5 Hz, 21H), 3.11 (s, 32H), 2.96 – 2.62 (m, 17H), 2.32 (s, 5H), 1.59 (d, J = 68.7 Hz, 78H), 1.33 – 1.10 (m, 41H).

Synthesis of Alexa-nanoSABER (Alexa-R_6AAN-alkyne-nitrile) with sequence Ac-Arg-Arg-Arg-Arg-Arg-Arg-Ala-Ala-Asn-Cys(StBu)-Pra-Lys(Alexa)-CBT:

Scheme S3. Synthetic route for Alexa-nanoSABER.

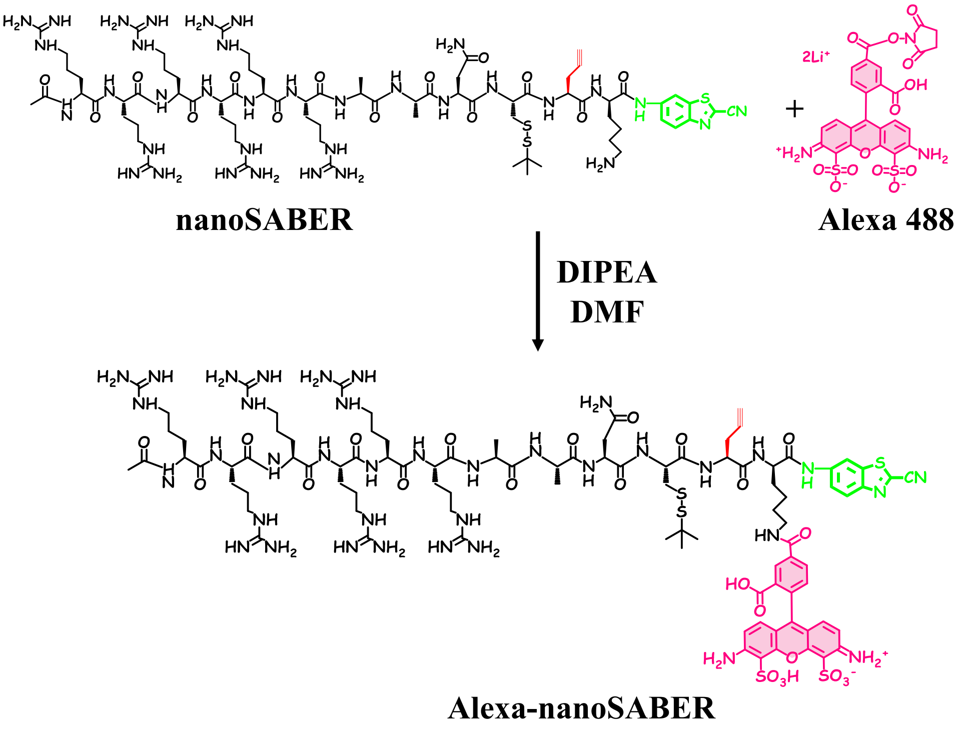

Synthesis of Alexa-nanoSABER: Alexa Fluor® 488 NHS ester (1 mg, 0.0015 mmol) solution with 10 uL diisopropylethylamine (DIPEA) was added to a solution of nanoSABER (10 mg, 0.005 mmol) in dry DMF, followed by 2 h stirring at RT in the dark. Alexa-nanoSABER was purified from the mixture using RP-HPLC with 50% yield. MS: calculated for Alexa-nanoSABER $[(M+H)^+]$: m/z 2341.68; observed MALDI-TOF/MS: m/z 2341.87 (Figure S11). The HPLC chromatogram of Alexa-nanoSABER and the corresponding UV-Vis spectra are shown in Figures S13a and S13b. The Raman spectrum of Alexa-nanoSABER is shown in Figure S13c.

^{1}H-NMR of Alexa-nanoSABER ($C_{95}H_{137}N_{37}O_{24}S_5^+$, d_6-DMSO, 400 MHz, Figure S12): δ 8.70 (d, J = 15.4 Hz, 11H), 8.33 (s, 1H), 8.16 – 7.97 (m, 43H), 7.79 – 7.55 (m, 13H), 6.93 (s, 462H), 6.68 (s, 1H), 6.48 (s, 14H), 4.25 (s, 1H), 4.18 (s, 54H), 3.38 (s, 26H), 3.33 (s, 286H), 3.04 (s,

70H), 2.91 – 2.62 (m, 1H), 2.62 (tt, J = 3.6, 1.8 Hz, 333H), 2.52 – 2.48 (m, 534H), 2.38 (ddd, J = 16.5, 6.0, 4.1 Hz, 789H), 1.85 – 1.75 (m, 73H), 1.72 – 1.50 (m, 5H), 1.42 (s, 41H), 1.26 – 1.05 (m, 404H).

2. Figures

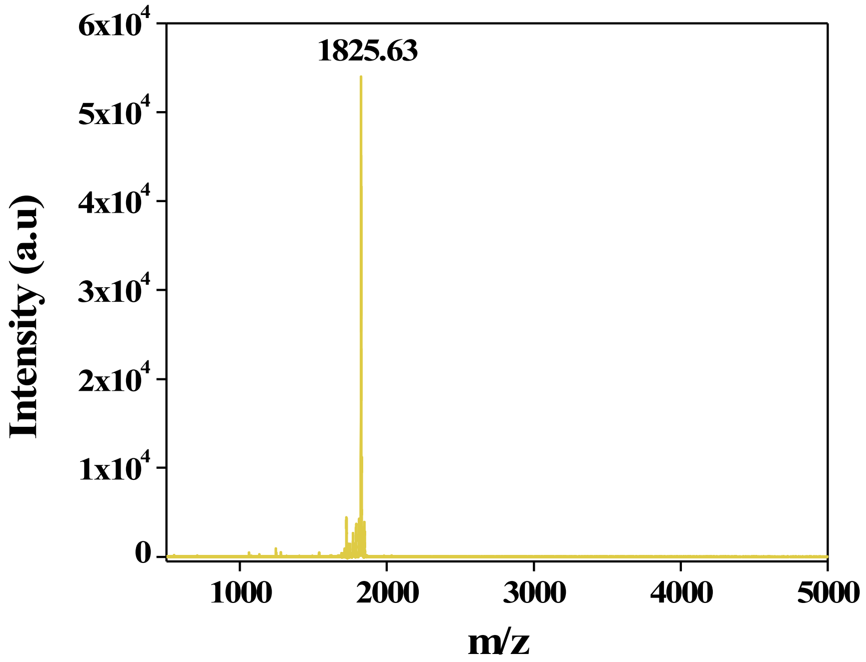

Figure S1. HR-MALDI-TOF/MS spectrum of nanoSABER. The spectrum reflects representative data from *in vitro* experiments repeated three times.

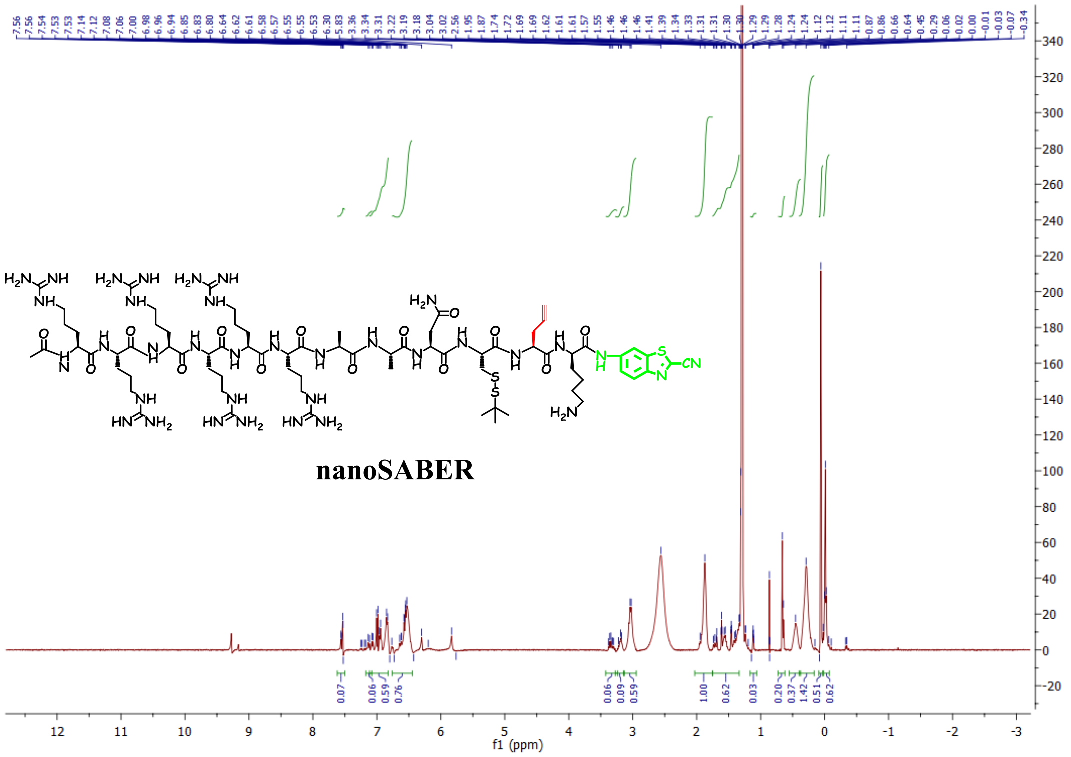

Figure S2. ^{1}H NMR spectrum of nanoSABER. The spectrum reflects representative data from *in vitro* experiments repeated two times.

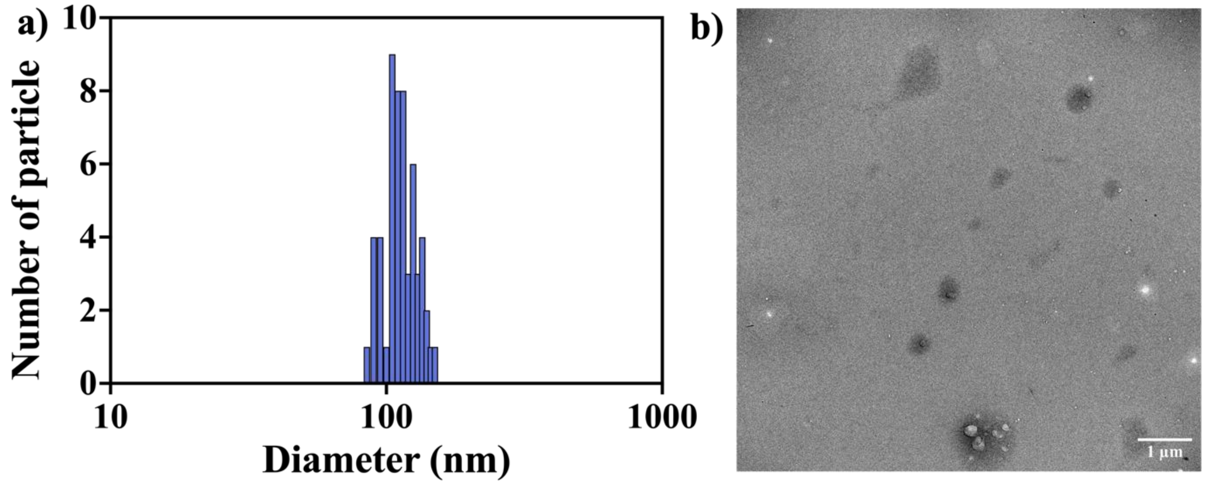

Figure S3. (a) Particle size distribution of alkyne-dimer nanoparticles as obtained from TEM. (b) TEM of nanoSABER solution showing the absence of self-assembled structures. Data are representative of three independent *in vitro* experiments.

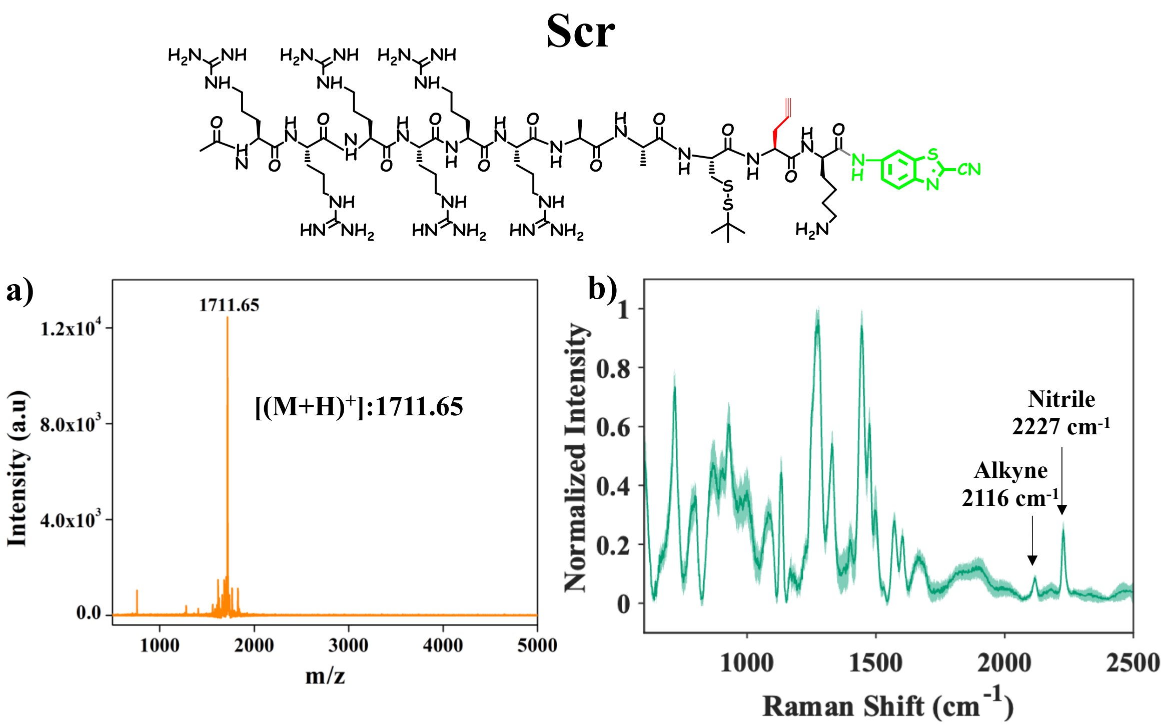

Figure S4. (a) HR-MALDI-TOF/MS spectrum and (b) Raman spectrum of Scr shown as mean±SD of 18 independent spectra. The spectra reflects representative data from *in vitro* experiments repeated three times.

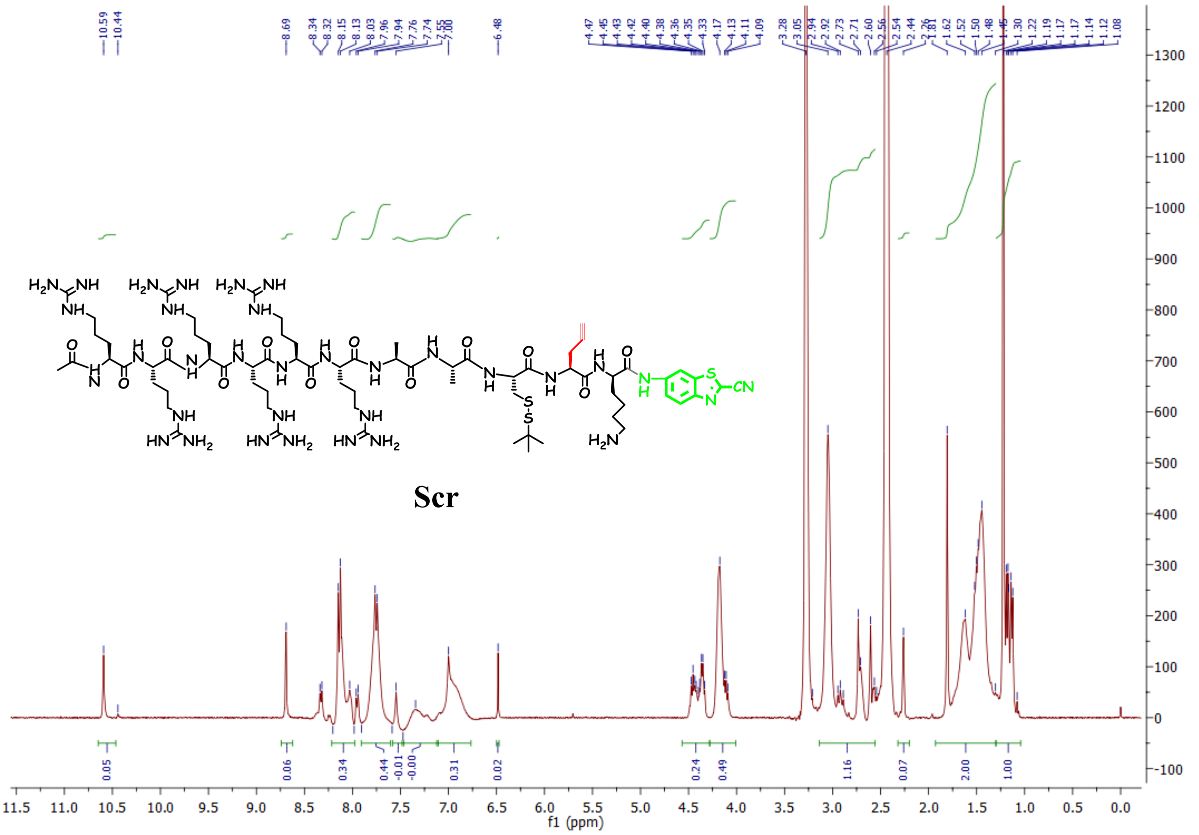

Figure S5. ^{1}H NMR spectrum of Scr. The spectrum reflects representative data from *in vitro* experiments repeated two times.

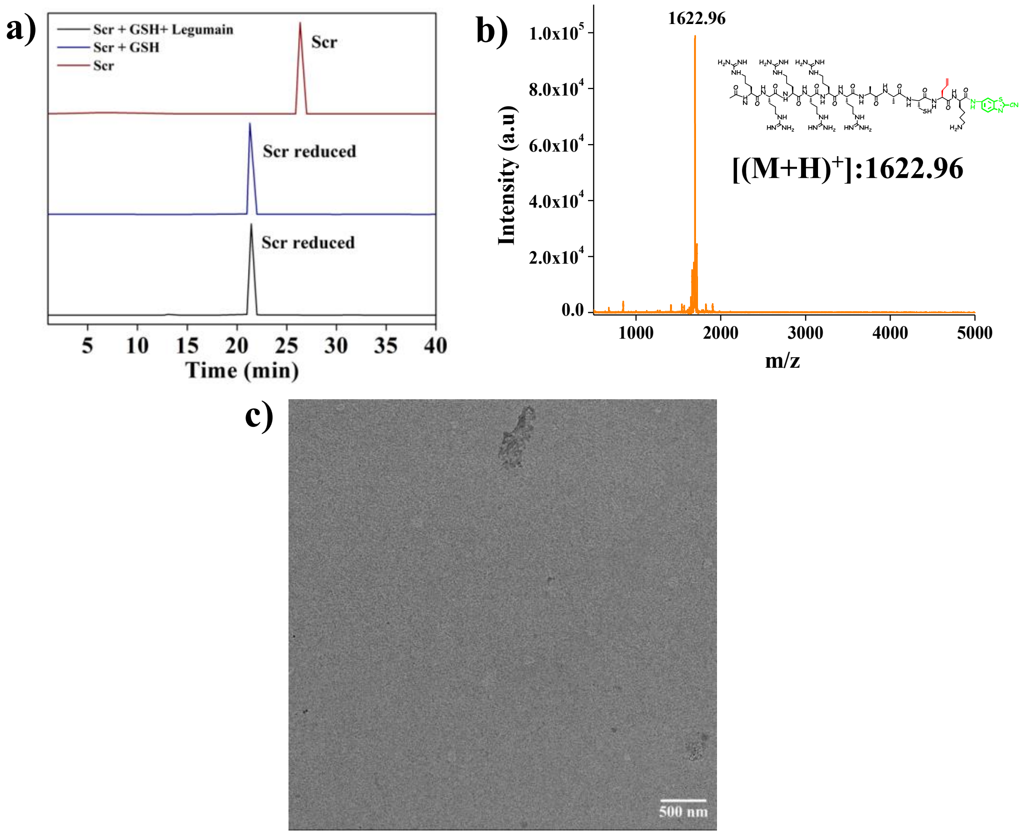

Figure S6. (a) HPLC chromatogram of 250 μM Scr (dark red) and 250 μM Scr + 1 mM GSH incubated for 3 h (blue), and 250 μM Scr + 1 mM GSH + 3 μL (10 μg/50 μL) legumain incubated for 3 h (black). (b and c) HR-MALDI-TOF/MS spectrum and TEM image of 250 μM Scr after incubation with 1 mM GSH and 3 μL (10 μg/50 μL) legumain for 3 h at 37 °C. The data shown are representative of three independent *in vitro* experiments.

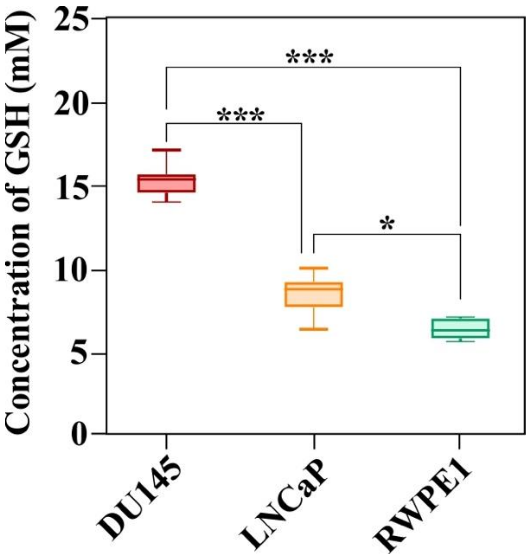

Figure S7. Intracellular GSH levels of DU145, LNCaP, and RWPE1 cells. DU145 cells have the highest levels of GSH, followed by LNCaP and RWPE1 cells. Data are shown as mean±s.d. (n=3 independent *in vitro* experiments). P-values of less than 0.001 (***), 0.001 to 0.01 (**) and 0.01 to 0.05 (*) were considered significant (one-way ANOVA with Tukey's HSD multiple comparison post-hoc test).

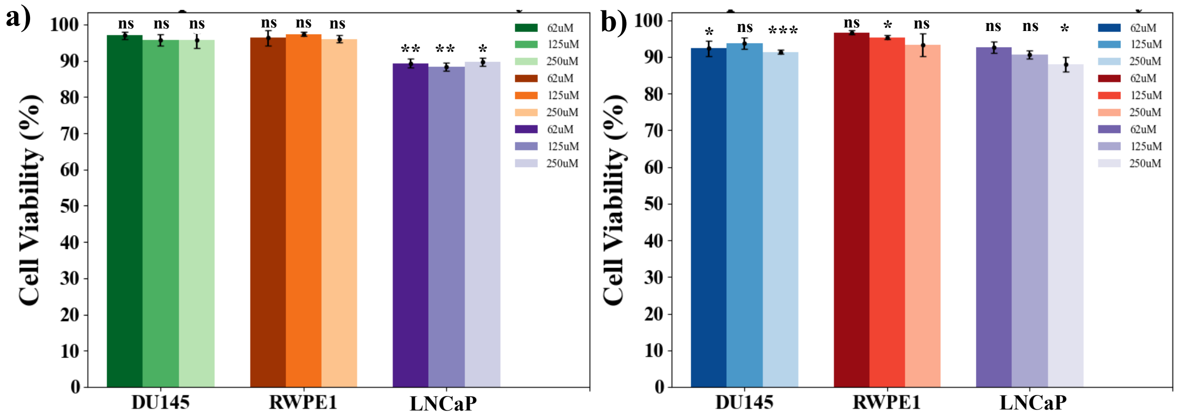

Figure S8. Cell viability of DU145, RWPE1, and LNCaP cells incubated with different concentrations of (a) R_6AAN-alkyne and (b) nanoSABER for 48 h. Data are shown as mean ± s.d. (n= 3 independent experiments); two-tailed Student's *t* test; *P < 0.05, **P < 0.01, and ***P < 0.001 vs. control (non-treated) groups.

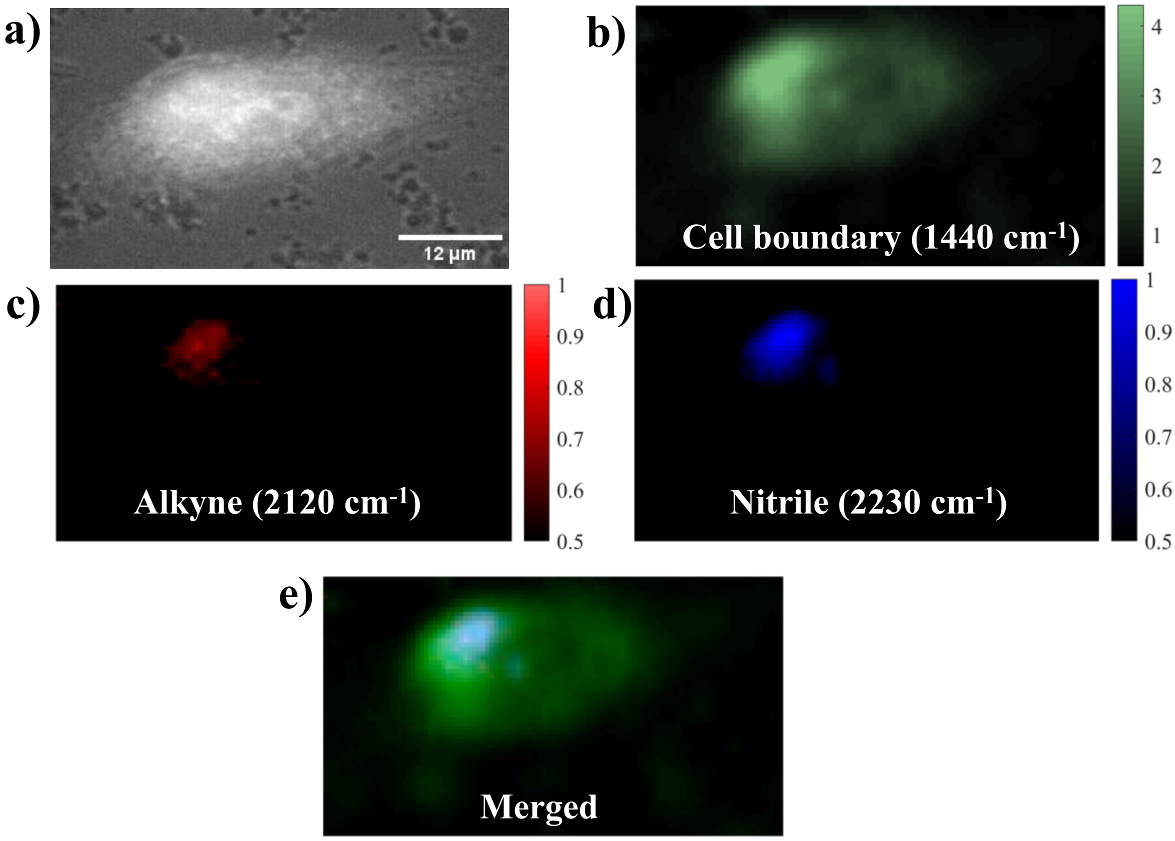

Figure S9. *In vitro* cellular Raman mapping. (a) Bright field and (b-d) Raman images recorded from DU145 cells incubated with 100 µM of Scr. (b) The 1440 cm^{-1} signal (green) corresponds to the CH_2 bending mode from intrinsic cellular components. The signals at (c) 2120 cm^{-1} (red) and (d) 2230 cm^{-1} (blue) correspond to the alkyne and nitrile signal, respectively, from the internalized Scr**.** (e) Merged image showing an overlay of endogenous cellular, alkyne, and nitrile Raman signals. The data is representative of n=3 independent measurements.

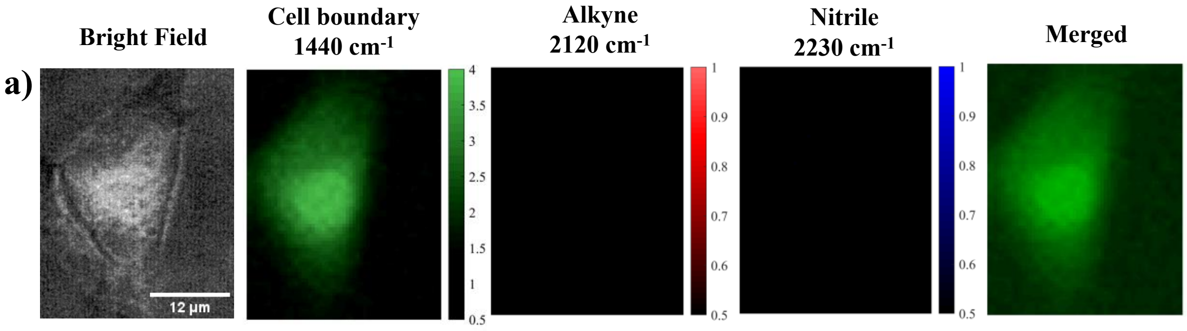

Figure S10. *In vitro* cellular Raman mapping of DU145 cells without any treatment. (a) Bright-field image of a DU145 cell. (b) The 1440 cm^{-1} signal (green) corresponds to the CH_2 bending mode from intrinsic cellular components. (c) and (d) Raman signal of alkyne and nitrile groups,

respectively. No signal corresponding to alkyne and nitrile groups was observed. The data are representative for n=3 independent experiments.

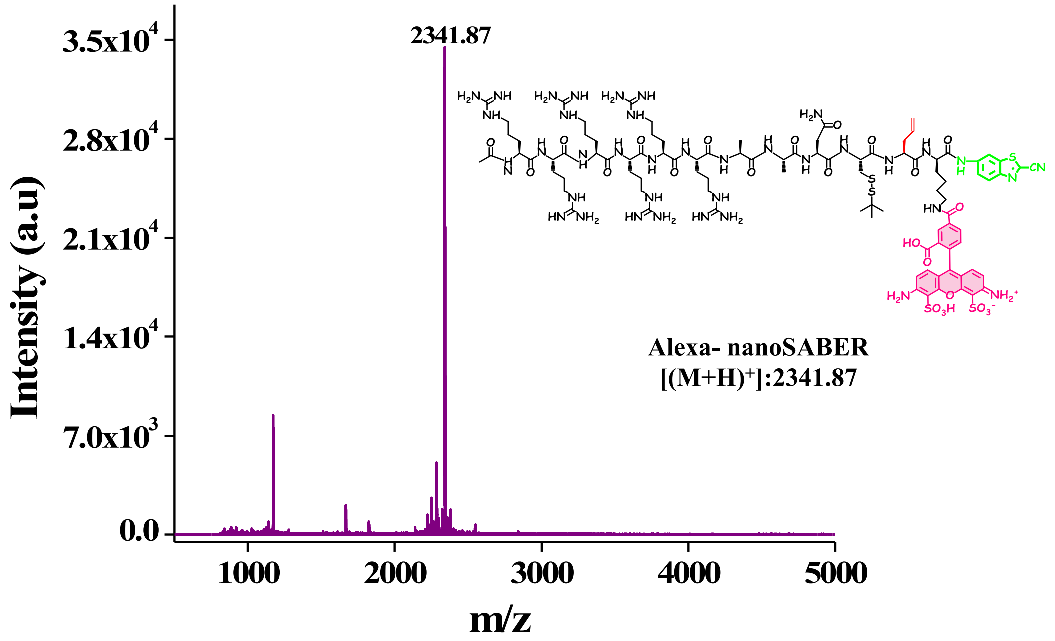

Figure S11. (a) HR-MALDI-TOF/MS spectrum of Alexa-nanoSABER. The spectrum reflects representative data from *in vitro* experiments repeated three times.

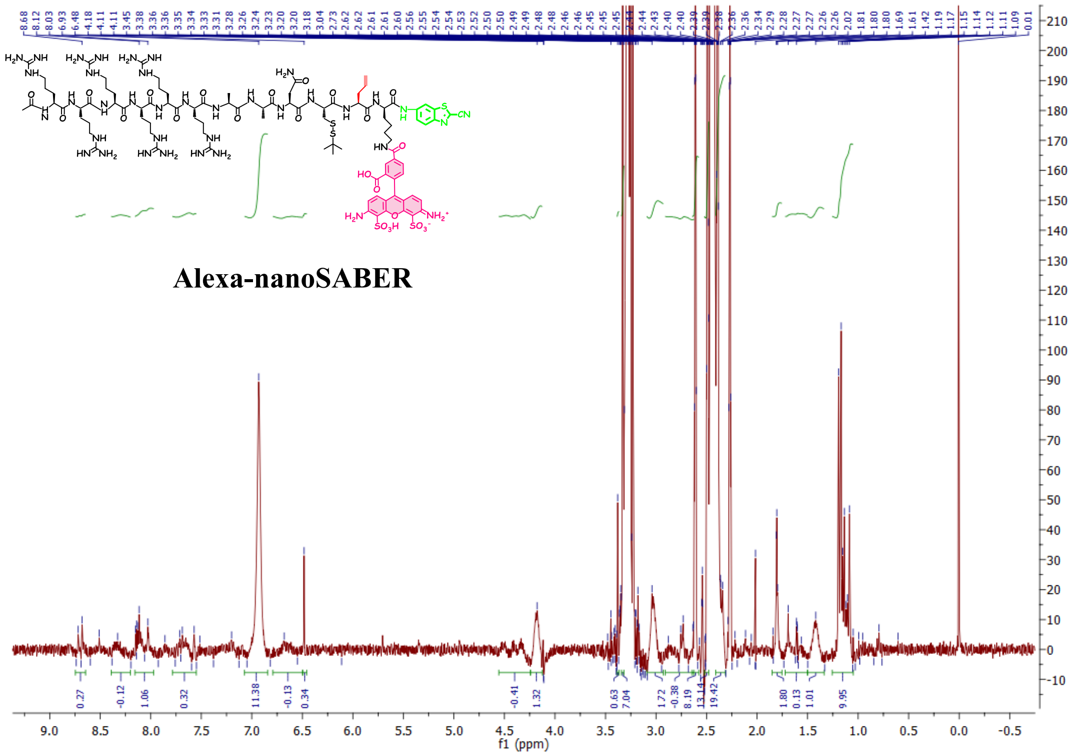

Figure S12. ^{1}H NMR spectrum of Alexa-nanoSABER. The spectrum reflects representative data from *in vitro* experiments repeated two times.

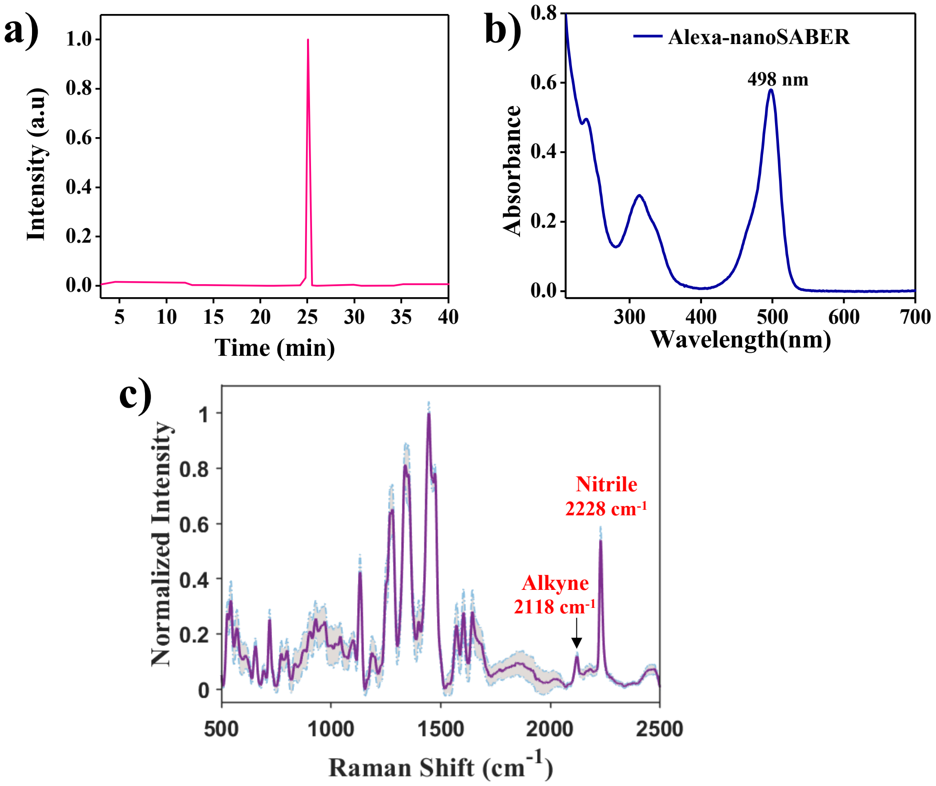

Figure S13. (a) HPLC chromatogram of Alexa-nanoSABER showing a retention time of 25 minutes and (b) corresponding UV-Vis spectrum with an absorbance maximum at 498 nm. (c) Raman spectrum of Alexa-nanoSABER showing the presence of alkyne and nitrile Raman peaks, shown as mean±SD (n=22 independent *in vitro* measurements). The HPLC chromatogram and UV-Vis spectrum represent data from *in vitro* experiments repeated two times.

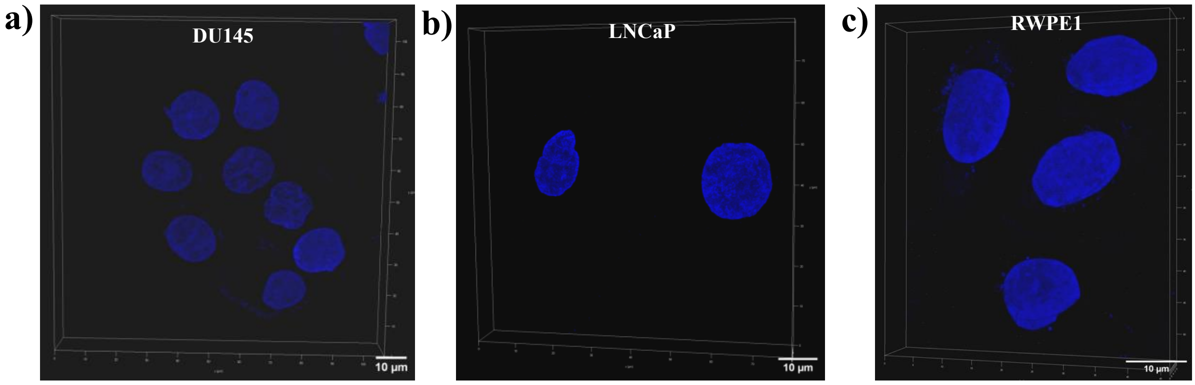

Figure S14. High-resolution confocal fluorescence images of a) DU145, b) LNCaP, and c) RWPE1 cells incubated with 3 μM Alexa 488 dye solution for 3 h. Cell nuclei were stained with DAPI (blue), and green fluorescence denotes a signal from Alexa 488 dye. All subpanels reflect representative images from *in vitro* experiments repeated three times.

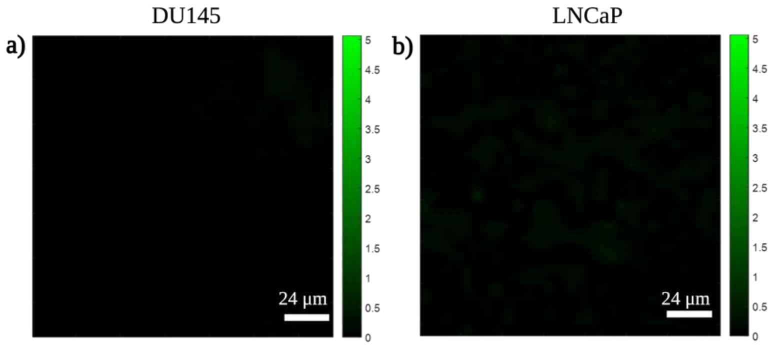

Figure S15. *In vivo* Raman imaging of (a) DU145 and (b) LNCaP tumor bearing mice 2 h post IV injection of PBS. Subpanels show representative data from three mice in each experimental group.

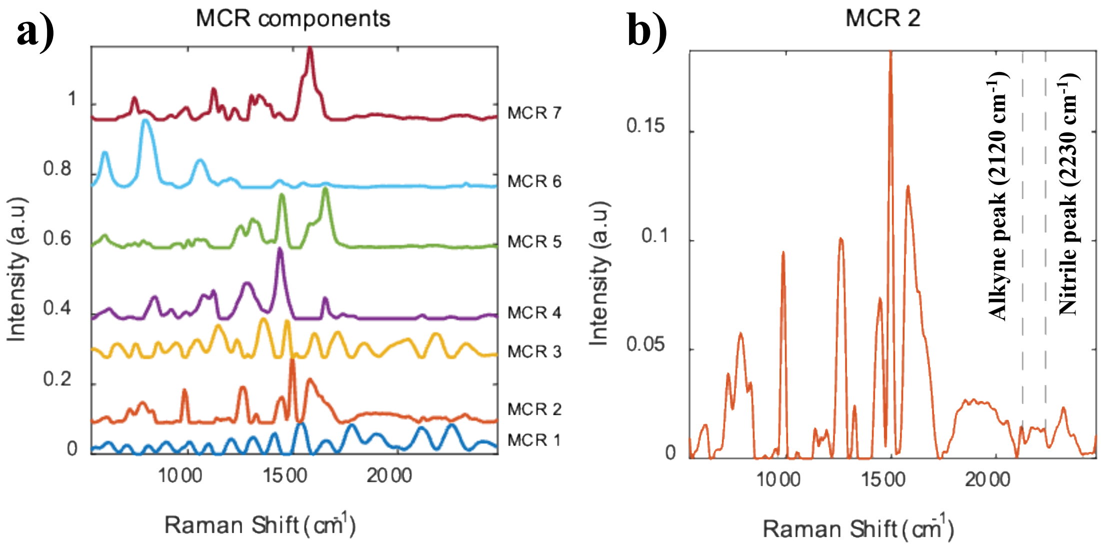

Figure S16. Multivariate Curve Resolution (MCR) was performed on the collected spectral matrix. MCR analysis breaks down the spectral matrix into a score and component matrix. Seven different MCR components were created. MCR1 has spectral features dominated by quartz, MCR2 is dominated by tissue-like spectra with alkyne/nitrile feature in the high wavenumber region, MCR3 is dominated by tissue-like spectra in the fingerprint region (with a few peaks in the high-wavenumber region) and MCR4-MCR7 have varied tissue-like

features. (b) Detailed analysis of the MCR2 component shows distinct alkyne and nitrile peaks, characteristic of the nanoSABER probe in the biologically silent spectral region. Data presented are obtained from three independent mice within each experimental group.
